# Artificial intelligence based advancements in nanomedicine for brain disorder management: an updated narrative review

**DOI:** 10.3389/fmed.2025.1599340

**Published:** 2025-05-13

**Authors:** Pankaj Dipankar, Diego Salazar, Elizabeth Dennard, Shanid Mohiyuddin, Quynh C. Nguyen

**Affiliations:** ^1^National Institute of Nursing Research, National Institutes of Health, Bethesda, MD, United States; ^2^School of Public Health, Epidemiology and Biostatistics, University of Maryland, College Park, MD, United States; ^3^Division of Hematology and Medical Oncology, Department of Medicine, Ellis Fischel Cancer Center, University of Missouri, Columbia, MO, United States

**Keywords:** artificial intelligence, brain disorders, deep learning, nanomaterial, machine learning, nanomedicine

## Abstract

Nanomedicines are nanoscale, biocompatible materials that offer promising alternatives to conventional treatment options for brain disorders. The recent technological developments in artificial intelligence (AI), particularly machine learning (ML) and deep learning (DL), are transforming the nanomedicine field by improving disease diagnosis, biomarker identification, prognostic assessment and disease monitoring, targeted drug delivery, and therapeutic intervention as well as contributing to computational and methodological developments. These advancements can be achieved by analysis of large clinical datasets and facilitating the design and optimization of nanomaterials for *in vivo* testing. Such advancement offers exciting possibilities for the improvement in the management of brain disorders, including brain cancer, Alzheimer’s disease, Parkinson’s disease, and multiple sclerosis, where early diagnosis, targeted delivery, and effective treatment strategies remain a great challenge. This review article provides an overview of recent advances in AI-based nanomedicine development to accelerate effective and quick diagnosis, biomarker identification, prognosis, drug delivery, methodological advancement and patient-specific therapies for managing brain disorders.

## 1 Introduction

Brain disorders are an umbrella term used to describe a set of serious illnesses that affect brain structure, functions, and often result in severe mental, emotional, and physical disabilities. Over the past several decades, brain related disorders have accounted for around 13% of all disease-related deaths worldwide, surpassing cardiovascular and cancer conditions. Additionally, brain disorders represent most of the all-cause mortality in Europe, defined by disability-adjusted life years (DALYs) (1). Also, Borlongan *et al.,* reported that the incidence of neurological disorders is becoming more prevalent and common in the United States (2). Certain brain disorders such as brain cancer, Alzheimer’s disease (AD), Parkinson’s disease (PD), and multiple sclerosis significantly contribute more to the global disease burden and are responsible for larger healthcare expenditures compared to other neurological conditions (3). Thus, we focus this narrative review on those brain disorders.

Each brain disorder is characterized by its unique pathophysiology, clinical symptoms, and treatment challenges. These challenges include unavailability of accurate, reliable and early diagnostic methods, treatment gaps, and corresponding patient costs (4–6). Thus, it is important to get better insight into these challenges and limitations for the development of effective management strategies to improve disease conditions. The most important challenge in treating Alzheimer’s and Parkinsons disease is ambiguity in diagnosis due to complicated etiology and pathological symptoms, prevent the early diagnosis and treatment (6). Apart from this, the development and release of new drugs into the market are affected due to lack of knowledge regarding specific biomarkers and molecular targets associated with these conditions. Thus, it is difficult to develop effective diagnosis and treatment strategies for the better management of brain related pathologies (7, 8). Another problem associated with such disorders is high-cost expenditures in their management. Previously, a research group conducted a study on Denmark based population to examine the trends of treatment expenditure on brain related disorders. Surprisingly, researchers found that the treatment cost is drastically increased in the case of comorbid patients dealing with brain related disorders. Also, the demand of healthcare resources is increased because patients having brain related disorders experience severe psychological distress that results into anxiety and depression (9). Additionally, anatomical structures such as blood–brain barrier (BBB) prevents the entry of large molecules and biologics, reduced the effectiveness of therapeutic drugs and makes treatment more challenging (10, 11).

Nanomedicines provide effective solutions to the above discussed challenges regarding management of brain related disorders. The nanomedicine exploits the concept of nanotechnology that can apply to manage different domains of disease including drug delivery, treatment, diagnosis and disease monitoring using nanostructures or nanomaterials (12). The selective nature of blood–brain-barrier, limit the delivery of large molecule biologics or therapeutic drugs into the affected regions of the brain. This problem can be addressed by utilizing nanocarriers to deliver the drugs that can easily cross the blood brain barrier (12). Also, the use of engineered nanoparticles enhances the bioavailability of drugs without any immune rection and cellular or tissue toxicity (10, 11).

In addition to this, the development of powerfully engineered multifunctional nanomaterials that offers the use of single smart nanomaterial to perform multiple functions including site specific drug delivery, conjugation of proteins or antibodies for detection and tagging with imaging molecules offers real time monitoring in *in vitro* and *in vivo* systems (13). Also, the nanocarriers are not only used for target delivery of drugs but also improving their pharmacokinetics that allows slow release of drug for extended periods and improved the therapeutic response in both Parkinson’s and Alzheimer’s patients (14). Moreover, the development of advanced neuro-imaging techniques such as functional MRI (f-MRI) and their integration with nanomedicines improves the diagnosis and treatment response. Although, standardization of their operating procedure in different environments, analysis and interpretation of produced complex images is another ongoing challenge (15, 16). Additionally, the technical limitations associated with the development of nanomedicines includes time consuming synthesis of nanomaterials or nanoparticles, lack of molecular understanding of their cellular or tissue toxicity and safety concern, clinical applications and challenge in manufacturing and commercialization (17). The technical challenges for the development and production of nanomedicine can be tackled through the application of artificial intelligence (AI) based approaches including machine learning and deep leaning methods (18).

Machine learning (ML) is a subset of artificial intelligence (AI) uses large volume of datasets to train the system and perform different task based on the training (19). The recent advancement in the machine learning algorithms and computing systems has widened their application in different disciplines including nanomedicine (20). The integration of AI and nanomedicine led to the transformation in different domain of disease management, starting from early diagnosis to therapeutics or treatment. Moreover, scientific community and pharma-based companies used machine learning based algorithms for the standardization and optimization of experimental conditions for the synthesis of novel nanoparticle or nanomaterial, prediction of drug delivery efficiency, and analysis of large clinical data to identify new targets that speed up manufacturing and commercialization of newly developed therapeutics (21, 22). Additionally, researchers used these complex algorithms to predict the behaviors of newly synthesized nanoparticles in *in vitro* and *in vivo* conditions to avoid or reduce the off-target effects by increasing targeting efficiency (23). Also, these models can potentially to analyze and extract the patterns from the complex images derived from advanced neuroimaging technique including functional magnetic resonance (fMRI). The analysis is helpful in identifying the minute changes in inner brain architectures critical for detection and classifying specific neurological disease conditions, identification of specific biomarkers, early disease diagnosis, personalized therapy, and prediction the clinical outcome (24–26).

Similarly, deep learning (DL) is a subset of machine learning that utilizes artificial neural network (ANN) with hidden multiple layers connected, and it can analyze text and image data to identify the patterns and perform different kinds of tasks such as prediction, classification and representations. The convolutional neural network (CNN) is capable of automatic processing and analysis of complex neuroimaging data that could be helpful for improving the diagnosis of brain cancer and other neurodegenerative diseases (92, 93). Also, it is very effective in the extraction image features that can be used for identifying minor alterations in brain regions and associated affected functions. Thus, this extraction features might be useful for predicting the disease progression, identification of disease-specific signatures (biomarker) for early diagnosis and personalized therapies for brain related pathologies (24, 27, 28).

In summary, applying AI-based methods including machine learning and deep learning advances the field of nanomedicine. These advancements have immense potential to improve different aspects of disease management ranging from diagnosis to treatments. The continuous evolution of these emerging technologies has led to the treatment of complex brain diseases of varying severity through the development of new therapeutics by exploiting the unique properties of nanomaterials. This review explores the applications of various AI based approaches that are currently used for the advancement of nanomedicine in the context of brain disorders and how adoption of these technologies improves the different aspects of disease management including diagnosis, biomarker identification, prognosis and disease monitoring, drug delivery and therapeutics and computational methodological advancements. Also, it will cover the technical challenges and limitations associated with them and lastly concluding remarks and prospects of this emerging technology.

## 2 Materials and methods

We conducted a comprehensive literature review to gather relevant studies for evidence synthesis and the detailed methodology employed for the search is described in the following sub-sections.

### 2.1 Study design

To ensure methodological rigor and transparent reporting, this comprehensive review was conducted following the Preferred Reporting Items for Systematic Reviews and Meta-Analysis (PRISMA) guidelines. This review explores the emerging field of integrating machine and deep learning techniques into nanomedicine. The primary focus is to synthesize evidence regarding the application of these techniques for the diagnosis, prognosis, biomarker identifications, and treatment of brain disorders, including brain cancer (glioblastoma and glioma), Alzheimer’s disease, Parkinson’s disease, and multiple sclerosis.

### 2.2 Inclusion and exclusion criteria

A comprehensive literature search was performed using the keywords and related MeSH (Medical Subject Headings) terms for “machine learning,” “deep learning,” and “nanomedicine” in the context of brain disorders. The inclusion criteria included original research on nanomedicine, machine learning, deep learning, and brain disorders. The detailed inclusion and exclusion criteria for the included studies are described in the subsequent section.

#### 2.2.1 Inclusion criteria

Included studies were original research focused on nanomedicine, machine learning, deep learning, and brain disorders. Below, we further describe the eligibility criteria for the included studies.

*Population characteristics:* Studies involving children and adults, with no restriction on gender, age, race, or ethnicity, were included. Studies that did not involve human participants, such as those utilizing murine models or molecular simulations, were also included.

*Intervention/Exposure:* Studies were included that show the application of machine and deep learning techniques in advancements of nanomedicine, theragnostic nanomedicine, and photothermal theragnostic for the management of Brain disorders (Brain cancer, Multiple sclerosis, Parkinson’s, and Alzheimer’s disease), but traditional, alternative, or complementary therapy-related studies are excluded. Computational modeling studies, including docking and molecular simulations, were incorporated if there was a direct relationship between machine learning/deep learning and nanomedicine.

*Outcome:* Studies that examined improving nanomedicine applications such as diagnosis, drug delivery, treatment, prognosis, disease monitoring, biomarker identification and diagnosis, and computational and methodological development by using machine learning, deep learning, or artificial intelligence were included.

*Study Characteristics and Design:* We included studies from all settings, such as communities, hospitals, specific healthcare facilities, and geographic locations. We included peer-reviewed original research articles.

#### 2.2.2 Exclusion criteria

The articles or studies are excluded if they were editorials, case reports, or interventions treatments that did not involve nanomedicine such as traditional or alternative or complementary therapies. Additionally, conference abstracts and dissertations, grey literature and non-English language articles were excluded from evidence synthesis.

### 2.3 Sources database and search strategy

The comprehensive literature search was conducted across electronic databases, including PubMed, Scopus, EMBASE, and Web of Science (Core Collection). Supplementary Table 1 describes detailed search strategies for each database. The search was limited to peer-reviewed articles published in English between 2014 and 2024.

### 2.4 Study selection

**Figure 1:**
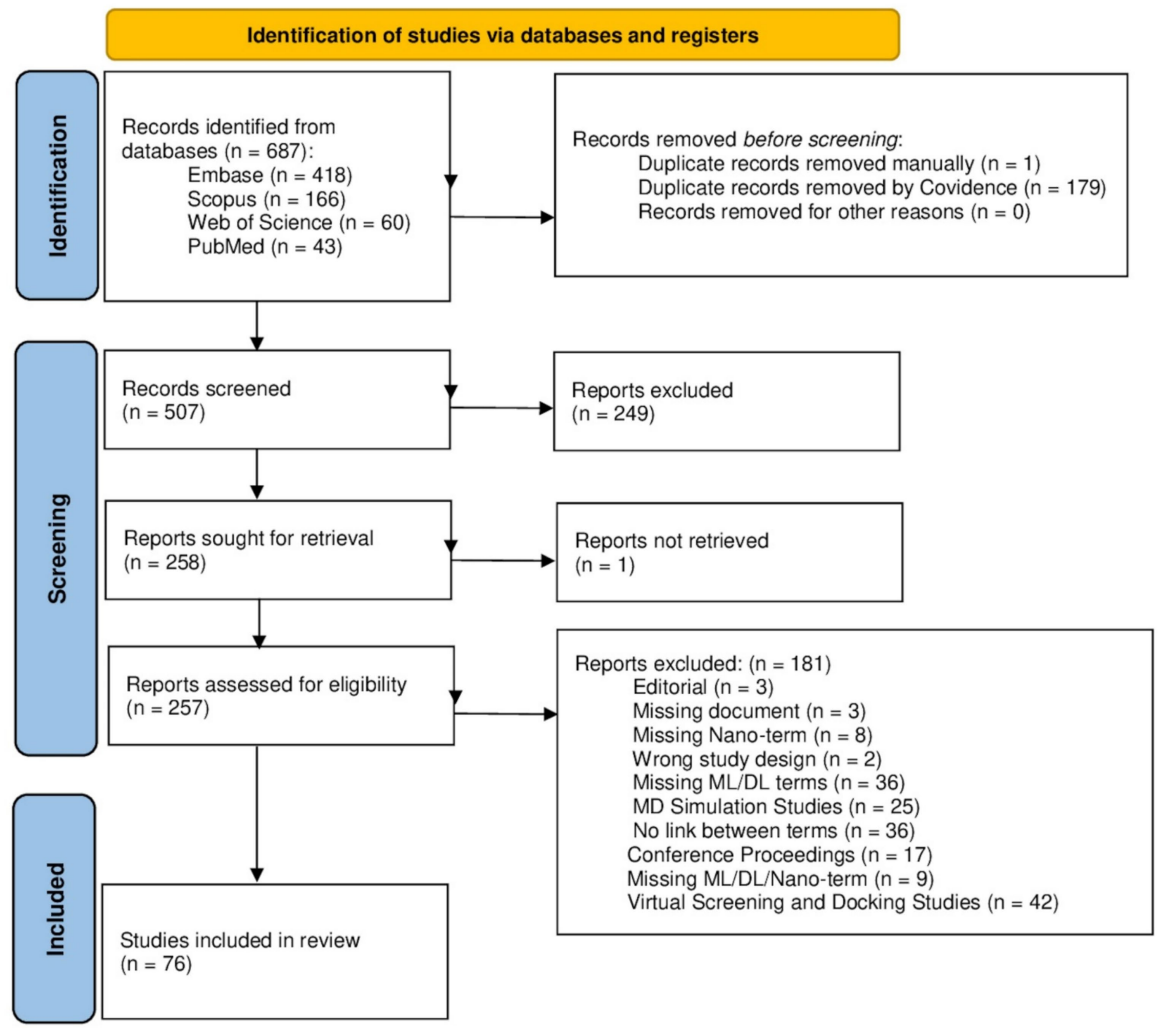
PRISMA 2020 flow diagram. This diagram depicts the flow of information through different stages of a literature review and maps the number of records identified, included, and excluded with detailed justifications.

Two independent reviewers conducted title-and abstract-based screening for the relevant studies. The eligible records were then accessed for full-text screening. Disagreements or conflicts were resolved through discussion. A PRISMA flow diagram displays the studies identified, screened, included, and excluded at each stage (Figure 1).

### 2.5 Data extraction

Data were extracted independently by two reviewers using a standard extraction template form with the following headings: general information (Title, Aim of Study), Methods (Study design, AI-based methods), Target disease, Participant Characteristics (population description), Study Results, and Study Implications. Any discrepancies in data extraction were resolved through consensus. EndNote 21.5 citation management tool was used to import and export citations and retrieve the full text of articles. Finally, Covidence software was used to remove duplicates and map the number of records identified, included, and excluded for the evidence synthesis.

## 3 Results

### 3.1 Selection of citations

A total of 687 articles were retrieved from different databases (PubMed = 43, Embase = 418, Scopus = 166, and Web of Science: Core collection = 60). After removing duplicates manually, only 507 records remained. Next, title and abstract screening was conducted, and 249 records were excluded due to ineligibility, and one record could not be retrieved. The remaining 257 records were assessed via full-text review to check eligibility, with 181 exclusions with different reasons mentioned in the PRISMA flowchart. Consequently, out of 76 studies, only 39 original research articles were used for data extraction and the remaining records comprising review articles and studies with insufficient data and lacking adequate quality or relevance were excluded (Supplementary Table 2). The results of the search and eligibility screening process are presented in Figure 1.

### 3.2 Historical evolution of AI powered strategies in nanomedicine for brain disorder management

**Figure 2:**
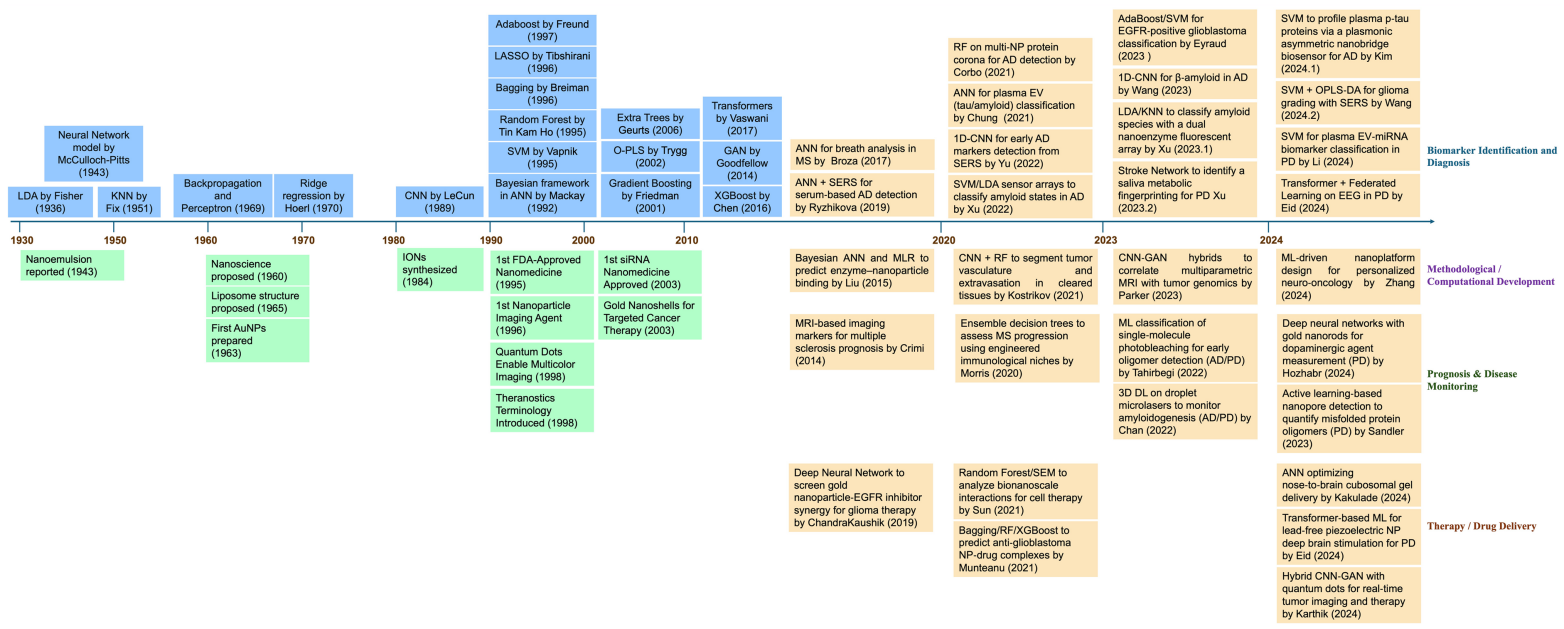
Timeline of important events related to research and development of nanomedicine, AI-based methods, and their applications in advancements of nanomedicine. This timeline (1930–2024) illustrates the progression of advancements in machine learning and deep learning methods as highlighted in blue boxes in the left upper panel. Additionally, the most relevant milestones in nanomedicine are highlighted in green boxes in the left lower panel. Since 2014, a growing integration between the two disciplines has been observed, especially in key areas such as biomarker identifications and diagnosis, methodological or computational development, prognosis, and therapy or drug delivery for the management of brain disorders are highlighted in orange box in the right upper and lower panel. AD, Alzheimer’s Disease; ANN, Artificial Neural Network; CNN, Convolutional Neural Network; DL, Deep Learning; EEG, Electroencephalogram; EGFR, Epidermal Growth Factor Receptor; EV, Extracellular Vesicles; GAN, Generative Adversarial Network; KNN, K-Nearest Neighbors; LDA, Linear Discriminant Analysis; ML, Machine Learning; MRI, Magnetic Resonance Imaging; MS, Multiple Sclerosis; NP, Nanoparticle; OPLS-DA, Orthogonal Partial Least Squares Discriminant Analysis; PD, Parkinson’s Disease; RF, Random Forest; SEM, Structural Equation Modeling; SERS, Surface-Enhanced Raman Spectroscopy; SVM, Support Vector Machine.

In the early 2000s, conventional machine-learning techniques were developed based on classical statistical methods. However, this ML model could not process multidimensional and complex neuroimage datasets. Several methods, such as SVM, have been used to analyze brain images or classify brain conditions. For example, several studies have employed SVMs to classify psychiatric conditions using input and neuroimage data generated from structural MRI (7, 8). In recent years, advanced neuroimaging techniques, such as diffusion tensor imaging (DTI). Moreover, functional MRI (fMRI) has made it possible to generate data in which ANN models have focused on processing images such as CNNs and transformer variants. This opens the possibility that these technological advances may identify biomarkers for pathologies such as Parkinson’s disease (PD), Alzheimer’s disease (AD), and Schizophrenia (29) (Figure 2).

Moreover, in the 2010s, introducing deep learning techniques was an important milestone. In general, the initial structures of the DL models resembled information processing in the brain, with neurons and layers of neurons processing different information. Now, the models are more complex and can identify and extract patterns from medical imaging (MRI), thus facilitating the detection of relevant features associated with the pathology under study (30). It is the case that CNNs can be used to analyze brain MRI images to identify structural and functional losses in Alzheimer’s disease and brain tumors. In addition, the feasibility of the existing multiple methods favors the use of different strategies, such as ensemble models, mixtures of experts, or hybrid models that combine the benefits of different models, for example, GAN and CNN (Figure 2).

Concurrently, scientists started applying the ML and DL techniques in the field of nanomedicine to design and optimize nanocarriers that could cross the selective blood–brain barrier to deliver the therapeutics to treat CNS-related disorders (31, 32). Also, advanced ML models are used to identify biomarkers for psychiatric conditions such as depression and anxiety using resting-state fMRI neuroimaging data (33). Others have employed transfer learning strategies to improve the diagnosis and classification of brain pathologies such as autism spectrum disorder. However, repositories of brain images and signals are sources of large-scale neuroimaging datasets with which ML/DL models can be trained and validated. Another field of action of ML models is deciphering mechanisms involved in brain disorders. For example, ML models have been used to understand brain connectivity associated with specific neurological conditions to identify specific therapies (34). These methods can help to predict more accurate clinical outcomes based on specific neuroimage signatures, making interventions more effective and personalized. Graph-based models and network analysis methods can be used to study brain networks and alterations related to CNS pathologies (35, 36).

In the case of personalized medicine, clinicians have used ML models to predict treatment responses in patients dealing with depression, maximizing their treatment efficacy (37). Recently, the focus on adopting ML/DL has become very interesting due to the benefits of using large volumes of data. Incorporating machine learning and deep learning models in the clinical setting enhances the diagnostic, prognostic, and treatment processes (38, 39). However, these implementations face several critical challenges, such as data privacy, mitigation of algorithmic biases, and limited interpretability of emerging machine learning models. Their implementation requires building trust among clinicians and establishing conditions that favor the large-scale adoption of deep and machine-learning technologies in diagnosing and treating neurological disorders (40, 41). Healthcare professionals know little about of the rationale behind the ML and DL-based prediction for disease prognosis and treatment strategies. Therefore, researchers from different backgrounds are trying to develop and implement an explainable AI system to generate universal and transparent models. On the other hand, using these models requires clear ethical guidelines to guarantee transparency and the correct development for their responsible use in neuroscience (42). The following sections discuss how integrating nanomedicine with machine and deep learning models has driven accelerated advances in the field. Nanotechnology enables detection at the nanometer scale, while the datasets generated by the various methodologies employed in nanomedicine benefit from the use of ML/DL models, which facilitate the identification of complex patterns and elevate the potential of nanomedicine to a new level.

### 3.3 Applications of advanced AI integrated nanomedicine for the management of brain disorders

**Figure 3:**
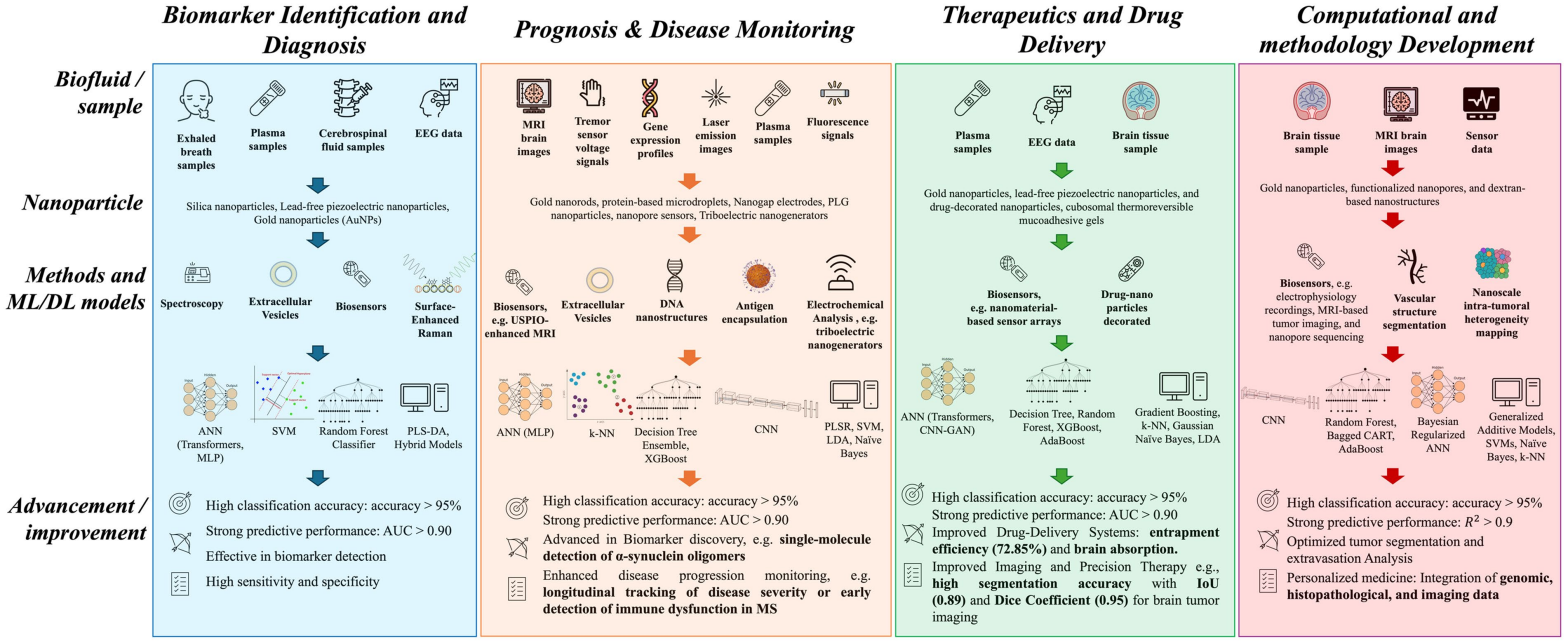
Summary of machine and deep learning methods and their applications in nanomedicine targeting brain disorders. This schematic diagram illustrates machine learning (ML) and deep learning (DL) applications in biomarker identification and diagnostics (Blue), prognosis and disease monitoring (Peach), therapeutic and drug delivery (Green) and computational and methodological development (Pink). Each column represents the workflow starting with different types of biological samples obtained from invasive to noninvasive methods, the latter being preferred because of their lower risk and greater accessibility. The use of nanoparticles allows high-sensitive detection levels and incorporating ML and DL methods lead to advancement of different facets of disease management. ANN, Artificial Neural Network; AUC, area under the ROC curve; CART, Classification and Regression Trees; CNN, Convolutional Neural Network; EEG, Electroencephalography; GAN, Generative Adversarial Network; IoU, Intersection over Union; k-NN, k-nearest neighbors; LDA, Linear Discriminant Analysis; MLP, Multi-Layer perceptron; MRI, Magnetic Resonance Imaging; MS, Multiple Sclerosis; PLGA, Poly (lactide-co-glycolide); PLS-DA, Partial Least Squares Discriminant Analysis; PLSR, Partial least squares regression; SVM, Support Vector Machine; USPIO, Ultrasmall superparamagnetic iron oxide.

The previous reports suggest that conventional treatment strategies are not effective for the management of brain disorders. The recent development in nanotechnology combined with AI based approaches has potential to overcome the challenges associated with traditional methods and has positive impact on the management of various brain disorders including, brain cancer, Alzheimer’s, Parkinson’s disease, and multiple sclerosis. These developments are observed in different disease management domains, including biomarker discovery for early diagnosis, prognosis and disease monitoring, therapeutics and drug delivery, and computational methodological developments. The following sub-section covers the detailed applications of AI based approaches in propelling nanomedicine forward and how they transform the various facets of brain disorders management (Figure 3).

#### 3.3.1 Biomarker identifications and diagnosis of brain disorders

The proper management of brain disorders depends on identifications of early biomarkers and diagnosis for timely interventions. The recent advancements in AI based nanomedicines revolutionize biomarker discovery and enable early diagnosis at a rapid pace and enable personalized healthcare for brain disorders. Surprisingly, there are no reliable biomarkers for Parkinson’s disease (PD) related cognitive decline or dementia. In this context the study done by Chung et al., demonstrated that plasma derived extracellular vesicle (EVs)-borne tau and *β*-amyloid have the potential to be used as a biomarker for Parkinson’s disease (PD). In this study, researchers isolated EVs from the blood (plasma) of PD patients of varying severity of disease ranging from mild to moderate stages and from control individuals and performed immunomagnetic reduction-based immunoassay to quantify the level of *α*-synuclein, tau, and Aβ1-42 proteins. Further, patient’s datasets considering attributes such as age, gender and differential expression of these EV markers were used to train the artificial neural network (ANN) model. Interestingly, this supervised model can predict the cognitive dysfunction related to Parkinson’s disease with an accuracy of 91.3%. Consequently, blood-plasma derived EV tau and Aβ1-42 emerged as important predictive biomarkers for early diagnosis and monitoring of PD derived cognitive decline or dementia (43) (Table 1).

**Table 1:** Summary of biomarker identifications and disease diagnosis based studies for the management of brain disorders.

S.N.	Study	Objective	Target disease	Body fluids	Core method	Nanomaterial	AI model
Biomarker identification							
1.	Chung et al. (43)	Use plasma EV-borne tau/amyloid to identify cognitive dysfunction in Parkinson’s disease	Parkinson’s disease	Blood (Plasma)	Immunomagnetic reduction-based immunoassay	Extracellular vesicles (EVs)	Artificial Neural Network (ANN)
2.	Resmi et al. (44)	Ultrasensitive, multiplexed SERS-immunoassay for detecting multiple Alzheimer’s Disease biomarkers (Aβ, tau)	Alzheimer’s disease	Blood (Plasma)	Multiplexed SERS-immunoassay platform	Spiky Star-shaped chitosan-coated gold nanoparticles (Cs-AuNPs)	Multilayer Perceptron (MLP), Radial Basis Function, Support Vector Machine (SVM), and Linear Discriminant Analysis (LDA)
3.	Yu et al. (45)	High-sensitivity cerebrospinal fluid approach combining SERS with a convolutional neural network for Alzheimer’s Disease (AD) detection	Alzheimer’s disease	Cerebrospinal fluid (CSF)	Surface-enhanced Raman spectroscopy (SERS)	Gold nanopyramid structure	1D - Convolutional Neural Network (CNN)
Disease diagnosis							
4.	Corbo et al. (46)	Develop a multi-nanoparticle protein corona approach to distinguish AD vs. healthy plasma samples for early AD detection	Alzheimer’s disease	Blood (Plasma)	Multi-nanoparticle protein corona profiling	Nano-platform (6 NPs) were 100 nm Silica (S) or Polystyrene (P) Nanoparticles with either Plain (P and S), amino-conjugated (P-NH2 and S-NH2), or carboxyl-conjugated (P-COOH and S-COOH)	Random Forest Classifier
5.	Etxebarria-Elezgarai et al. (47)	Surface-Enhanced Raman Spectroscopy (SERS) of cerebrospinal fluid to detect Alzheimer’s at preclinical/prodromal stages	Alzheimer’s disease	Cerebrospinal fluid (CSF) fractions	Surface-enhanced Raman spectroscopy (SERS)	Gold nanoparticles (Au NPs) substrates	Partial Least Squares Discriminant Analysis (PLS-DA)
6.	Meehan et al. (48)	Identify aptamer-based plasma markers (Aptamarkers) for brain amyloid deposition associated with AD	Alzheimer’s disease	Blood (Plasma)	Neomer library	Aptamer	Extra Trees, Random Forest, Gradient Boosting, and Logistic Regression
7.	Ryzhikova et al. (49)	A SERS-based blood test for AD, distinguishing mild/moderate AD and other dementias from healthy controls	Alzheimer’s disease	Blood (Serum)	Surface-Enhanced Raman Spectroscopy (SERS)	Silver colloidal nanoparticle (Ag NPs)	Artificial Neural Network (ANN) with Genetic Algorithm Feature Selection
8.	Wang et al. (50)	GO/Au-based SERS detection of Aβ1–42 for early AD diagnosis	Alzheimer’s disease	Blood (serum) (synthetic Aβ1–42 solutions)	Surface-enhanced raman spectroscopy (SERS)	Graphene oxide/gold nanohybrids	1D Convolutional Neural Network (1DCNN), and Support Vector Machine (SVM)
9.	Wang et al. (61)	Ultrasound-enriched colorimetric lateral flow assay (LFA) for tau-protein detection in AD	Alzheimer’s disease	Plasma or serum	Colorimetric lateral flow assay (LFA) with ultrasound enrichment	Colloidal gold nanoparticles (AuNPs)	k-Nearest Neighbor (KNN), and Gaussian Process Regression (GPR)
10.	Xu et al. (52)	ML-assisted dendrimer-based fluorescent sensor array to identify different aggregation states of Aβ for AD diagnosis	Alzheimer’s disease	Serum/CSF	Fluorescent sensor array	Pyrene modified fifth generation polyamidoamine (G5-PAMAM) dendrimers	Linear Discriminant Analysis, Decision Tree, SVM, and Logistic Regression
11.	Xu et al. (54)	Machine learning–assisted dual-enzyme sensor array (nanoenzyme and bioenzyme) to classify amyloid species relevant to AD	Alzheimer’s disease	Blood (Plasma)	Dual-enzyme (Nanoenzyme + Bioenzyme) Fluorescent Sensor Array	Nonoenzyme (AuNPs) and Bioenzyme (Horseradish Peroxidase)	Linear Discriminant Analysis and k-Nearest Neighbor (KNN)
12.	Xu et al. (53)	Saliva metabolic fingerprinting for Parkinson’s Disease diagnosis via nanoparticle-enhanced LDI-MS	Parkinson’s disease	Saliva	Laser desorption–ionization mass spectrometry (LDI-MS)	Nanoparticle-enhanced laser desorption–ionization mass spectrometry	LASSO, XGBoost, SVM, Random Forest, and Specialized “Stroke Network”
13.	Eid et al. (55)	ML-powered, lead-free piezoelectric nanoparticle-based DBS system for diagnosing/evaluating PD	Parkinson’s Disease	Brain Tissue	Deep brain stimulation (DBS)	Lead-free piezoelectric nanoparticle	Transformer Networks + Hybrid Simulated Annealing–Particle Swarm Optimization (SA-PSO), and Federated Learning
14.	Li et al. (56)	Identify plasma EV-derived miRNA biomarkers to distinguish idiopathic REM sleep behavior disorder vs. PD vs. healthy controls	Parkinson’s disease	Blood (Plasma)	Next Generation Sequencing (NGS)	Plasma EV-derived miRNA	Support Vector Machine (SVM)
15.	Broza et al. (57)	Develop a rapid, noninvasive breath test using nanomaterial-based sensors for diagnosing Multiple Sclerosis (MS)	Multiple sclerosis	Exhaled Breath	Gas Chromatography–Mass Spectrometry (GC–MS)	Nanomaterial-based sensor array	Multilayer Perceptron–type (MLP-type), and ANN
16.	Eyraud et al. (58)	Plasma nanoDSF denaturation profiles for EGFR classification in glioblastoma (GBM)	Brain cancer	Blood (Plasma)	Nano differential scanning fluorimetry (nanoDSF)	Plasma nanoDSF denaturation profile	SVM, Random Forest, and AdaBoost, Logistic Regression (with LOOCV)
17.	Rani et al. (59)	Nanoscale imaging technique combined deep learning for rapid MRI-based brain tumor detection	Brain cancer	Brain tissue	Nanoscale MRI imaging	Nanotechnology based detection scheme (NBDS) (gold nanoparticle and quantum dots)	Deep Neural Network
18.	Sun et al. (60)	Ratiometric SERS approach to quantify glioma cells intraoperatively and guide surgical resection	Brain cancer	Glioma cell	Surface-enhanced raman spectroscopy (SERS)	Silver nanoparticles (AgNPs)	Polynomial Regression Model, and Artificial Neural Network
19.	Wang et al. (51)	SERS-based distinction of gliomas at cellular and tissue levels	Brain cancer	Glioma tumor cells	Surface-enhanced Raman spectroscopy (SERS)	Gold Nanoshell (SiO_2_@Au) particles and Gold Nanoisland (AuNI)	SVM, and Orthogonal Partial Least Squares Discriminant Analysis (OPLS-DA)

Similarly, the conventional diagnostic methods lack early detection of Alzheimer’s disease (AD) because of the absence of reliable biomarkers. In this context, Resmi et al., proposed solution for early detection of Alzheimer’s Disease (AD) using an ultrasensitive SERS-based immunoassay integrated with machine learning approach. This technique utilized an aluminum SERS substrate to detect key AD based biomarkers such as Aβ40, Aβ42, p-tau and t-tau in the blood plasma with sensitivity of attomolar levels. Next, the author used these biomarkers-based datasets and feeds to different machine learning models (MLP, Radial Basis Function, SVM, LDA) to classify and differentiate between mild cognitive impairment (MCI), AD patients, and healthy individuals (44). In conclusion, the development of this AI integrated SERS technique enables the early diagnosis of Alzheimer’s disease by detecting these biomarkers with high precision and accuracy. Like the previous report, Yu *et al.,* suggest that combining SERS with deep learning model (CNN) to analyze cerebrospinal fluid to identify new biomarkers for Alzheimer’s disease (AD). The CNN models are used to analyze CSF based datasets and they can distinguish AD patients with healthy individuals with an overall performance of 92% while 100% for normal individuals, and 88.9% for AD patients. Interestingly, The CNN based classifications are strongly corelated with the clinical dementia rating (CDR) (45). Therefore, this new hybrid technique (SERS and CNN) has potential to identify biomarkers of Alzheimer’s disease with high precision and accuracy detection (Table 1). Thus, the integration of AI with nanotechnology is promising development for the discovery of new biomarkers associated with brain disorders, which is critical for early diagnosis and timely intervention for better disease outcomes.

Furthermore, the integration of AI-inspired machine and deep learning techniques with nanomedicines transforms the diagnosis of neurodegenerative diseases like Alzheimer’s and Parkinson’s, multiple sclerosis and brain cancer. Importantly, AI-based models can be trained by feeding large clinical datasets and these models have potential to analyze large datasets, identifying patterns, and perform extraction of these patterns to make predictions leading to earlier and more accurate diagnoses, crucial for timely interventions and improved patient outcomes. For instance, Corbo et al., developed a non-invasive diagnostic platform for the early detection of Alzheimer’s disease. Notably, this platform utilized a multi-nanoparticle protein corona signature is used to detect a minute change in human plasma protein signatures. Further, these datasets are analyzed by machine learning model including random forest to distinguished plasma samples of healthy and disease subjects with >92% specificity and ≈100% sensitivity, demonstrating its potential for of Alzheimer’s disease (46). Similarly, Etxebarria-Elezgarai et al., used Surface-enhanced Raman Spectroscopy (SERS) with gold nanoparticles (AuNPs) to analyze cerebrospinal fluid samples from Alzheimer’s disease (AD) patients. Combining SERS with AI-based partial least square discriminant analysis (PLS-DA) achieved 100% accuracy in classifying early-stage AD patients compared to 85% for the healthy control group (47) (Table 1).

Importantly, brain amyloid deposition is the hallmark of Alzheimer’s disease, which can be used for diagnosis of Alzheimer’s disease (AD). A newly developed aptamer-based plasma test identifies brain amyloid deposition and, when integrated with a machine learning model, the ExtraTreeClassifier improved the accuracy and predictive power with sensitivity of 0.88, specificity of 0.76 and AUC of 0.79. the overall reports suggest that this method is capable of diagnosis and monitoring the progression of Alzheimer’s disease (48). Interestingly, another scientific group utilized a modified SERS technique with the combination of multivariate statistical methods for the diagnosis of Alzheimer’s disease by analyzing blood serum. In this method researchers used colloidal silver nanoparticles (Ag NPs) as an active SERS substrate.

Furthermore, artificial neural networks (ANNs) were used to analyze SERS spectra to differentiate Alzheimer’s disease (AD) subjects from healthy control in binary model with sensitivity of ≈ 96% and achieved diagnostic sensitivity of 98% for differentiating Alzheimer’s disease (AD) individuals, Healthy control (HC), and Other Dementia (OD) samples in a tertiary model. This study highlights that combination of SERS and ANN models has potential for accurate and early Alzheimer’s disease detection (49). Also, another group used similar concept for the development of modified SERS technique, which involves, combining SERS with AI-based models to enhance detection of Aβ 1–42 monomers, which is important pathological biomarker of Alzheimer’s disease. This SERS platform contains graphene oxide/gold nanohybrids (GO/AuNPs) for the detection of Aβ 1–42 monomers and fibrils form with detection limits of 0.0232 and 0.0192 ng mL^−1^, respectively.

Next, scientist applied support vector machine (SVM) and one-dimensional convolutional neural network (1D-CNN) algorithms to analyze fibril orientation for accurate diagnosis of Alzheimer’s disease (50). Conventional colorimetric lateral flow assay (LFA) utilizes colloidal gold nanoparticles (AuNPs) for the diagnosis of Alzheimer’s disease, which exhibits low specificity and sensitivity. Importantly, the study led by Wang *et al.*, proposed an advanced method that exploits the machine learning algorithms with optimized colorimetric LFA, and ultrasound enrichment led to detection of tau proteins in undiluted blood serum samples with improved sensitivity. Various machine learning algorithms were used for different purposes such as k-nearest neighbor (KNN) for classification as well as Gaussian process regression (GPR) for accurate quantification of tau protein with enhanced classification, prediction accuracy of 98.11 and 99.99%, respectively, and a limit-of-detection (LOD) of 10.30 pg. mL^−1^. Thus, this improved method has potential to detect tau protein for clinical diagnosis of Alzheimer’s disease (AD) with greater sensitivity and precision (51) (Table 1).

Additionally, another research group advanced methods involving fluorescent sensor arrays through the integration of machine learning based algorithms including linear discriminant analysis (LDA) for the diagnosis of Alzheimer’s disease (AD). Notably, the sensor array consists of pyrene modified fifth generation poly-amidoamine (G5-PAMAM) dendrimers that allow parallel detection of 11 A*β*40/Aβ42 aggregates including monomer, oligomer and fibril forms present in different interferants, serum media and cerebrospinal fluid (CSF) with enhanced accuracy of 100%. The integration of LDA with fluorescent sensor array has tremendous potential for the early Alzheimer’s disease diagnosis with greater accuracy and precision (52). Similarly, Xu *et al.,* developed another fluorescent sensor array with dual coupling of nonoenzyme (AuNPs) and bio enzyme (horseradish peroxidase) for the detection of ultralow *β*-amyloid (Aβ) proteins (monomer, oligomer and fibril form) in blood plasma of Alzheimer’s induced mouse model. Further, machine learning algorithms such as k-nearest neighbor (KNN) and linear discriminant analysis (LDA) were employed to identify various β-amyloids aggregates and can distinguish Alzheimer’s induced mouse model and healthy mice with 100% accuracy (53).

The following section explores how the recent synergy of AI driven methods with nano-diagnostic techniques led to the advancements in the diagnosis of Parkinson’s disease. Recently, Xu *et al.,* developed a non-invasive diagnostic method using human saliva samples analyzed with deep learning-based nanoparticle-enhanced laser desorption–ionization mass spectrometry. This approach successfully mapped the saliva metabolic fingerprint helps to differentiate between healthy subjects and Parkinson’s disease participants with an AUC of 0.8496 (54). Moreover, deep brain stimulation (DBS) has been used to treat Parkinson’s disease, but the traditional use of lead electrode poses a risk of metal toxicity. To address this, scientist have developed a new approach called Lead-free piezoelectric nanoparticle-based DBS (LF-PND-DBS). This innovative method utilizes lead free piezoelectric nanoparticle to deliver electrical stimulation and thus eliminating the risk of metal toxicity (Table 1).

Recently, Eid et al., further advanced this technology by incorporating machine learning techniques such as transformer networks, hybrid simulated annealing–particle swarm optimization (SA-PSO), federated learning using an optimized LF-PND-DBS system. In a cohort patients study this optimized system achieved high accuracy (99.1%) and specificity (98.2%) for diagnosis and demonstrating its potential for precise diagnosis and personalized treatment delivery (55). Furthermore, another interesting study done by Li et al., developed a non-invasive method for diagnosing Parkinson’s disease using plasma blood samples. The authors have analyzed small molecule miRNA associated with extracellular vesicles (EVs) in the plasma of healthy and diseased patients by applying support vector machine (SVM) model. This algorithm can effectively identify EVs miRNA signatures and has potential to distinguish between plasma samples from healthy control and those with Parkinson’s disease individuals with an AUC of 0.916 (56). In conclusion the integration of AI driven techniques with nano-diagnostic tools substantially improved the accuracy, specificity, sensitivity, and predictive abilities leading to precise classification and early detection of the Alzheimer’s and Parkinson’s diseases pave the way for timely intervention and improved patient outcomes (Table 1).

Multiple sclerosis is a chronic neurodegenerative disease and it is conventionally diagnosed with the help of invasive methods. To overcome these challenges researchers are actively pursuing non-invasive solutions, particularly through the integration of AI and nano-diagnostic techniques. In this context, the study led by Broza et al., developed a rapid, non-invasive technique analyzing volatile organic compounds (VOCs) in breath samples using gas chromatography–mass spectrometry (GC–MS) and a nanomaterial-based sensor array and built a predictive model with an artificial neural network (ANN). GC–MS was used for analyzing distinct VOC profiles in MS patients compared to controls. The combination of sensor array and artificial neural network can easily differentiate between MS patients from heathy control group with accuracy of 90%. Also, the blind validation was carried out and it can differentiate other studied groups with great accuracy such as 95% PPV for MS remission versus control group with 100% sensitivity and 100% NPV for MS non-treated versus control groups, and 86% NPV for relapse versus controls (57). In conclusion, AI powered GC–MS methods have potential to diagnose multiple sclerosis with improved accuracy (Table 1).

Furthermore, the integration of AI based models with core techniques utilizing nanoparticle or nanomaterial has improved the diagnosis of brain cancer. In this context, Eyruad et al., utilized nano differential scanning fluorimetry (nanoDSF) to generate plasma denaturation profiles (PDPs) from the blood plasma of brain cancer patients and healthy individual’s subjects. PDPs are further analyzed by AI based algorithms to detect altered EGFR expression profile and perform automated classification with accuracy of 81.5% (58). Thus, this technique can be useful for the better diagnosis of brain cancer associated with alteration of epidermal growth factor receptor (EGFR). Importantly, Rani et al., utilized nanoscale imaging with deep neural network-based segmentation to locate tumor in precise manner in MRI based images, which cannot be possible by using conventional methods. The integration of neural networks with MRI can easily locate tumors with a high accuracy of 97.3% (59). Moreover, the complete surgical removal of brain tumors is essential to prevent the risk of glioma recurrence, but the invasive nature of tumor cells makes surgery challenging due to limitations in identifying tumor boundaries or margins.

To address this limitation Sun et al., introduced modified surface-enhanced Raman scattering (SERS) technique for detection of glioma cells using silver nanoparticles (AgNPs). Further, the SERS produced spectral peaks are analyzed using artificial neural networks and polynomial regression modeling. The peak around 655 and 717 cm^−1^, correspond to glioma cell proportion and can estimate the glioma cell percentages in simulated sample and frozen sample with R^2^ of 0.98 and 0.85, respectively. Therefore, the integration of AI based algorithms with SERS would be useful for identification of tumor margins in fresh tissue samples and it could be helpful for real time visualization of tumor boundaries during intraoperative brain tumor surgery (60).

Similarly, Wang et al., introduced the concept of differentiating glioma at both cellular and tissue levels. To make a distinction at the cellular level between normal astrocytes and non-central nervous system (CNS) tumor cells, the researchers used a modified SERS technique utilizing gold nanoshell (SiO_2_@Au) particles and support vector machine (SVM) algorithms. Subsequently, gold nanoisland (AuNI) SERS substrates used for detection of glioma tumor tissue and further employing support vector machine (SVM) and orthogonal partial least squares discriminant analysis (OPLS-DA) to distinguish glioma and trauma tissues, identification of various tumor grades, and determination of IDH mutation with great accuracy and precision. These findings indicate the feasibility of SERS-based methods for both single-cell and tissue-level GBM detection, with potential for real-time intraoperative diagnosis (61). In conclusion the integration of AI driven techniques with nano diagnostic tools significantly improved the accuracy, predictive abilities leading to precise classification and detection of the glioma Versus normal cells demonstrating the revolution of the field of brain cancer diagnosis (Table 1).

#### 3.3.2 Prognosis and disease monitoring

The management of brain related disorders also depends on prognosis and monitoring the severity of disease in regular intervals of time. Currently, the recent developments and progress in AI based nanomaterials are used to track the disease by monitoring the expression level of specific biomarkers, neurotransmitters release, and analyzing image datasets (Table 2). This section will explore how different nanomaterials and AI based approaches are used for prognosis and disease monitoring of brain related pathologies.

**Table 2:** Summary of Prognosis and Disease Monitoring Based Studies for the Management of Brain Disorders.

S.N.	Study	Objective	Target disease	Sample/Analytes/Symptoms	Core method	Nanomaterial	AI model
1.	Sandler et al. (62)	Single molecule nanopore approach to detect misfolded protein oligomers, aiding PD drug discovery	Parkinson’s disease	Misfolded protein oligomers	Solid-state nanopores and DNA barcoding	DNA nanostructure	Active Learning–Based Docking Simulations for Small Molecule Inhibitor Optimization
2.	Tahirbegi et al. (63)	High-throughput single-molecule method with ML to detect oligomers of alpha-synuclein and amyloid beta	Alzheimer’s disease and Parkinson’s disease	Amyloidogenic proteins	Single-molecule photobleaching (SMP) and total internal reflection fluorescence microscopy	Gold nanoparticles (Au NPs)	SVM, and Multilayer Perceptron (MLP)
3.	Chan et al. (64)	3D deep-learning strategy to track amyloidogenesis (applicable to AD or PD) using microlaser imaging	Alzheimer’s disease and Parkinson’s disease	Amyloidogenic proteins	Protein-based microdroplet laser array	Amyloid nanostructure conformation	Multi-convolution architecture (2D + 1D), plus fully connected layers
4.	Hozhabr et al. (65)	A colorimetric sensor with gold nanorods to discriminate dopaminergic agents relevant to PD progression monitoring	Parkinson’s disease	L-DOPA, Carbidopa, Benserazide	Colorimetric Sensor	Gold nanorods	Linear Discriminant Analysis (LDA), and Partial Least Squares Regression (PLSR)
5.	Komoto et al. (66)	Time-resolved detection of neurotransmitters in mouse brain tissue for PD diagnostics	Parkinson’s disease	Dopamine, serotonin, norepinephrine	Multicolor sensor	Nanogap electrodes	XGBoost, and Random Forest Classifiers
6.	Muraoka et al. (67)	Proteomic profiling of plasma EVs to identify potential CTE biomarkers in former NFL players	Alzheimer’s disease	Blood (Plasma) EVs	Proteomic profiling	Extracellular vesicles (EVs)	Linear Discriminant Analysis (LDA), Naive Bayes, and SVM
7.	Crimi et al. (68)	Imaging markers in early multiple sclerosis for disease prognosis and better treatment	Multiple sclerosis	Monoamine neurotransmitter	MRI scan	Ultrasmall super paramagnetic iron oxide (USPIO) and gadolinium (Gd)	XGBoost
8.	Kim et al. (69)	Self-powered tremor sensor classifying Parkinson’s disease tremor severity.	Parkinson’s disease	Tremor	Tremor sensor	Catechol-chitosan-diatom hydrogel (CCDHG)	Linear SVM, and KNN
9.	Morris et al. (70)	Biomaterial-based “immunological niches” for real-time monitoring of multiple sclerosis relapse/treatment efficacy	Multiple sclerosis	T-cells	Adoptive transfer of encephalitogenic T-cells	Antigen encapsulating PLG nanoparticles	Bagged Tree, and Singular Value Decomposition (SVD)

In both Alzheimer’s and Parkinson’s diseases, it is evident that the accumulation of oligomers plays a critical role in the neurodegeneration process. Aβ and *α*-synuclein are the key proteins involved in these conditions. Therefore, it is essential to develop high-throughput methods for quantitatively estimating these oligomers with high accuracy and precision. To address this problem Sandler *et al.,* proposed a method that combined solid-state nanopores and DNA barcoding to detect and quantify *α*-synuclein oligomers. Additionally, researchers developed a multiplexed detection system to detect and analyze multiple samples or different conditions within a single experiment. The nanopore sensing detection systems measured change in ionic current based on the analyzed proteins size, shape, and charge. Also, it allows the testing of drugs that inhibit oligomer agglomeration. Researchers employed the combination of molecular docking and iterative active learning to identify small molecule inhibitors for *α*-synuclein aggregation. Furthermore, these potential drugs were identified using machine learning models that continuously updated and refined their predictions as new experimental data were generated. As a result of this the two potential inhibitors (Anle-138b and I3.08) were identified, which can inhibit *α*-synuclein’s secondary nucleation (62) (Table 2).

Another strategy for studying the aggregation of these oligomers is to develop methods capable of early detection of this process. Tahirbegi et al., proposed a single-molecule photobleaching (SMP) technique to detect early-stage aggregation of Aβ40 and α-synuclein. This method outperforms conventional techniques that struggle to identify the transition from monomer to low-order oligomer at low physiological protein concentrations. Typically, proteins are immobilized on a solid surface to ensure single-molecule resolution. In the case of α-synuclein samples, these samples were collected after 12 h of incubation with various concentrations of gold nanoparticles. These nanoparticles function as aggregation modulators, exhibiting inhibitory and promotional effects depending on their concentration. Subsequently, Total Internal Reflection Fluorescence microscopy recorded fluorescence intensity over time. The data obtained from the fluorescence was used to train SVM and MLP models. Overall, the MLP model achieved an accuracy of 83.5%, with a relaxed true positive rate (TPR) of 98.9% (63). The AI-driven advancements in nano diagnosis methods enable the potential to monitor the progression of Alzheimer’s and Parkinson’s diseases (Table 2).

The study, by Chan et al., utilized a peptide-encapsulated droplet micro laser and a 3D deep learning strategy such as multi-convolution architectures (2D + 1D), along with fully connected layers to detect minute nano structural spectral shifts during amyloidogenesis and to monitor their progression. Interestingly, this multimodal approach achieved high accuracy of over 95% for all kinds of datasets including training, validation, and test sets and demonstrating AI based nanomaterials have the potential to its potential for the progression of these neurodegenerative diseases (64). Furthermore, the integration of AI and nanotechnology enables us to develop strategies to detect and track the changes in neurotransmitters. In this context, Hozhabr et al., developed a multicolor sensor using gold nanorods to detect and monitor dopaminergic drugs (L-DOPA, carbidopa, benserazide). Researchers employed the linear discriminant analysis (LDA) and partial least squares regression (PLSR) to process high-dimensional data for enhanced detection with achieved 100% accuracy in classification and quantification tasks and detection limits ranging from 0.03 micromolL^−1^ to 0.9 micromolL^−1^ (65).

Moreover, other methods have integrated the detection of neurotransmitters in brain tissue which is demonstrated by Komoto et al., who report that nanogaps and XGBoost are used to measure tunneling currents to detect specific neurotransmitters. XGBoost served as a qualifier for differentiating neurotransmitters. Although the metrics were not satisfactory (F1-score 0.52), the single-molecule measurement enabled high-resolution monoamine neurotransmitter detection in both solutions and mouse brain tissue (66). One type of nanoscale biomarker is extracellular vesicles (EVs), which are implicated in Alzheimer’s and other tauopathies. These vesicles have been found to transport tau and amyloid-beta oligomers between brain cells. In the Muraoke et al., study, plasma samples were collected from NFL players and age-matched controls to isolate EVs using size-exclusion chromatography, followed by proteomics analysis with mass spectrometry. The study also evaluated total tau (t-tau) and phosphorylated tau (p-tau181) levels using a modified, ultrasensitive immunoassay. These protein levels were used to train models, including linear discriminant analysis, Naive Bayes, and SVM, to differentiate between NFL players and the control group with achieved accuracy of 85% and AUC of 0.85. The finding suggests that these EVs contained tau proteins are associated with the progression of Alzheimer’s disease (67) (Table 2).

Besides detecting proteins, imaging is also utilized to track the biomarkers in neurodegenerative diseases such as multiple sclerosis and Parkinson’s. Similar to this concept, Crimi et al., introduced machine learning-based classification framework to identify spatiotemporal patterns of lesions observed in multiple sclerosis. In this study, the author used ultrasmall superparamagnetic iron oxide (USPIO)-enhanced MRI to highlight the macrophages activity. Further different clustering methods such as K-means, hierarchical clustering, Gaussian mixture models (GMM), and spectral clustering were applied for a two-tier classification framework, including lesion classification and for patient classification.

Furthermore, scientists developed a tremor sensor to monitor the Parkinson’s disease (PD) severity and treatment response by tracking tremor patterns of patients in a real-time manner (68). The study led by Kim et al., worked on this concept and introduced a stretchable and self-healable catechol-chitosan-diatom hydrogel (CCDHG) as a novel biocompatible electrode for triboelectric nanogenerators (TENGs) which can be utilized as a tremor sensor to detect low-frequency vibrations in PD patients. Additionally, scientist applied machine learning algorithms such as linear SVM and KNN that enables sensor to classify the tremors based on different tremor intensities with an accuracy of 100% (69). Similarly, monitoring disease progression of multiple sclerosis by employing nanomaterial is reported by Morris et al., in this study authors implanted microporous poly (*ε*-caprolactone) (PCL) scaffolds subcutaneously in SJL/J mice to examine the immune cell activity and gene expression pattern at different stages of the disease (pre-symptomatic, symptomatic, remission, relapse). Further, a Bagged tree algorithm was used to predict the disease state and relapse by analyzing changes in gene signatures. This minimally invasive method allows us to study both the disease’s mechanism and the responses to treatments in MS and other immune diseases (70) (Table 2).

#### 3.3.3 Therapeutics and drug delivery

This section explores the recent development in AI based nanomedicine led to transformational changes in drug delivery methods and treatment strategies for the management of brain disorders. Also, covers different machine and deep learning strategies that improves pattern recognition and extraction from the different datasets.

The conventional machine learning approaches used input features as a static value, but the study done by Munteanu et al., presents a novel approach using perturbation theory machine learning (PTML), which dynamically adjusts molecular descriptors, such as logP, by incorporating perturbations observed across different experimental conditions (e.g., cell types) and then uses these values as input to train the ML model. The authors used molecular descriptors of the drug, the nanoparticles, experimental conditions, and the perturbations to which the system is exposed. Despite testing approximately 800,000 drug-nanoparticle complexes, no pair of clinical interest is mentioned; however, among the relevant features that were found, given that the best model was a decision tree-based method, are Perturbation of Polar Surface Area in different cell types, perturbation of logP (lipophilicity) in other cells, nanoparticle surface area of acceptor atoms, nanoparticle size in various experimental conditions, Van der Waals volume of nanoparticles in different situations, and nanoparticle polarizability in other conditions. This shows that drug solubility and nanoparticle physicochemical properties in predicting cytotoxicity are essential in predicting a cytotoxic complex and collaborate in the design of new treatments (71) (Table 3).

**Table 3:** Summary of therapy and drug delivery based studies for the management of brain disorders.

S.N.	Study	Objective	Target disease	Therapeutics agent	Application	Nanomaterial	AI model
1.	Munteanu et al. (71)	Predict anti-glioblastoma efficacy of drug-decorated nanoparticles using molecular descriptors	Brain cancer	Drug–nanoparticle combinations	Anti-glioblastoma efficacy	Drug-decorated nanoparticles (DDNPs)	Perturbation Theory Machine Learning (PTML), and Multiple Classifiers (Random Forest, Bagging, AdaBoost)
2.	Chandra Kaushik et al. (72)	Investigate the synergistic antitumor efficiency of anti-EGFR-iRGD protein with gold nanoparticles (AuNPs) for glioma	Brain cancer	Anti-EGFR-iRGD and AuNP complex	Anti-tumor efficiency	Gold nanoparticles (AuNPs)	Deep Neural Networks (DNN)
3.	Karthik et al. (73)	Combine quantum dots (QDs) with a real-time imaging-guided therapeutic system to refine radiotherapy targeting	Brain cancer	Real-time imaging-guided therapeutics (RIGT)	Advanced tumor segmentation	Combine quantum dots (QDs)	Hybrid CNN–GAN
4.	Kakulade et al. (74)	Formulate an intranasal selegiline HCl–loaded cubosomal gel to enhance bioavailability and brain targeting	Parkinson’s and Alzheimer’s	Selegiline HCl–loaded cubosomal thermoreversible mucoadhesive gel	Controlled drug release	Cubosomes	Artificial Neural Network (ANN)
5.	Sun et al. (75)	Explore 1 T-MoS2 nanosheets to enhance neuronal cell fate and therapy, modulating biomolecular interactions for potential PD treatments.	Parkinson’s disease	1 T-MoS2 (octahedral coordination) and 2H-MoS2 (triangular prism coordination) with fibronectin and liposomes	Neuronal cell fate therapy	1 T-MoS2 nanosheets	Polynomial Regression Model and Artificial Neural Network

Additionally, advances in glioblastoma and glioma have increasingly focused on identifying specific molecular targets for therapeutic intervention. The epidermal growth factor receptor, or EGFR, is a tyrosine kinase receptor widely studied in cancer. The mutation in EGFR gene is responsible for dysregulated protein expression results in abnormal cellular functions including survival, proliferation, differentiation and migration. In this context, Chandra Kaushik et al., conducted *in silico* study that applied deep learning, molecular docking, time course simulation and synthetic biology to identify the nanoparticle which has synergistic antitumor activity with anti-EGFR-iRGD protein. In this study, author employed deep learning models were used to predict potential nanoparticle which has higher binding affinity with maximum inhibitory potential against EGFR-iRGD protein using PubChem chemical compound library. The predictions suggest that Gold Nanoparticles (AuNPs) were the most effective inhibitors of EGFR. Further, to validate this finding researcher performed molecular docking study to determine the binding affinity and found that AuNPs and EGFR have a binding affinity of (−3.5 kcal/mol). Additionally, the authors applied synthetic biology to examine the effects of EGFR and AuNPs interaction in downstream signaling pathways including Ras/Raf/mitogen-activated protein kinase pathway, the PI3K/Akt pathway, the PLCγ pathway, and the STAT3 pathway by applying Boolean circuit analysis. Through this analysis, the researchers validated the impact of AuNPs (0.45 mmol) on EGFR downstream signaling pathways over time, and found that AuNPs gradually diminish EGFR activation, which results in inactivation of downstream players leads to tumor suppression. Overall, results suggest that antitumor efficiency of anti-EGFR-iRGD protein with gold nanoparticles (AuNPs) complex is effective against EGFR driven tumors (72). Thus, this study opened the door to predicting and validating nanoparticle-based therapies that target specific downstream molecular pathways, providing a new basis for developing effective precision medicine (Table 3).

Moreover, there is a significant advancements in imaging techniques are also reported, which is evident from the study conducted by Karthik *et al.*, who introduced a hybrid CNN-GAN model to improve tumor segmentation and refine radiotherapy targeting through quantum dots (QDs). In this approach the quantum dots provide high-resolution contrast at varying tissue depths, thereby improving the performance of real-time MRI while CNN-GAN hybrid model substantially improved the extraction and segmentation of brain tumors. CNN contributes to feature identification by recognizing distinct patterns in healthy and tumor tissue while determining characteristics such as size, shape, and heterogeneity. Meanwhile, GAN utilizes a generator-discriminator framework that refines the segmented images to capture specific tumor details. The researchers used high-resolution 3D MRI brain scans to segment the tumors and compare their findings with the ground truth, achieving a Dice Coefficient of 0.95, which confirms high segmentation precision. The results indicate a notable advancement in neuro-oncology, as it allows for the proposal of real-time and accurate patient treatment (73) (Table 3).

While nanomedicine and AI have made significant strides in cancer treatment by optimizing drug delivery and imaging precision, similar approaches have also been directed toward Parkinson’s and Alzheimer’s diseases. This field focuses on enhancing drug bioavailability, penetrating the blood–brain barrier, and targeting neuromodulation. Specifically, machine learning and deep learning models are utilized to improve drug treatments and brain stimulation. In the realm of neuromodulation, as discussed in the Diagnostic/Biomarkers section, Eid *et al.,* proposed integrating machine learning methods to diagnose and evaluate treatment efficacy using lead-free piezoelectric nanoparticle-based deep brain stimulation (LF-PND-DBS). They trained machine learning models using EEG datasets from Kaggle and clinical records of Parkinson’s to optimize DBS stimulation parameters. In this instance, the best-performing model was based on transformers hybridized with Simulated Annealing and Particle Swarm Optimization, producing very promising metrics; for example, the F1 score achieved a value of 99.2. This study paves the way for advancements in neuromodulation without requiring invasive techniques, minimizing adverse effects, and integrating personalized treatment (55) (Table 3).

Among the treatments for the symptoms of Parkinson’s disease, there are also drugs such as selegiline HCl, which works alongside levodopa to slow the progression of Parkinson’s symptoms. Many drugs have difficulty crossing the blood–brain barrier (BBB), making it essential to find different dosage forms that enhance drug bioavailability. In this context, Kakulade et al., developed, characterized and evaluated the pharmacokinetics of selegiline HCl (SGH)-loaded cubosomal thermoreversible mucoadhesive gel for nose-to-brain drug delivery. The formulations utilized cubosomal gel as a drug delivery vehicle which can transpassing the blood brain barrier (BBB) and carrying both hydrophobic and hydrophilic drug. In this study, artificial neural network was used to optimize formulation by considering various parameters including formulation variables (glycerol monooleate, Poloxamer 407, Tween 80) and output variables (particle size, entrapment efficiency (EE), and drug release). The study showed this AI based approach optimized nanoparticle size with controlled drug release for up to 6 h, and a cumulative drug delivery rate in the cubosomes exceeding 70% with 1.90-fold increase in pharmacokinetic performance in brain compared to the drug solution alone (74). Similar study done by Sun *et al.*, explore the effect of molybdenum disulfide (MoS₂) nanosheets and MoS₂ crystal structure on cellular functions including adhesion, differentiation, and neuroprotection. These findings revealed that the octahedral conformation of 1 T-MoS₂ nanosheets can to regulate biomolecular interactions, resulting in enhanced neuroprotection. Further, researchers employed random forest analysis to determine the important variables to quantify the relationship between the nano structural architectures. Lastly, structural equation modeling (SEM) confirmed a causal relationship, showing that neurite outgrowth directly related with increased cell survival. Finally, the researchers discovered that MoS₂ laminas can alter the folding of alpha-synuclein, thus preventing its aggregation. Molecular dynamics simulations showed that 1 T-MoS₂ interacts with *α*-synuclein fibrils, destabilizing *β*-sheet structures and facilitating fibril disaggregation. In a Parkinson’s disease mouse model, direct striatal injection of MoS₂ nanosheets enhanced memory retention and reduced neuroinflammation. Therefore, this nanosheet has therapeutic potential against Parkinson’s disease (75) (Table 3).

#### 3.3.4 Methodological and computational development

Applying machine learning and deep learning models in nanomedicine permeates different fields beyond the clinical area. Advances in brain disorders include the nanoscale study of other molecular components, image analysis in the study of tissues, and even molecular interactions. By combining AI and nanomedicine, researchers are overcoming traditional methods, challenges and limitations, offering more accurate and cost-effective solutions. This section explores recent computational advances in nanoparticle interactions, imaging analysis, and molecular sequencing. As we have observed up to this point, integrating AI and nanotechnology drives throughput clinical precision, increases data granularity, and improves diagnostic accuracy. AI methods’ ability to extract patterns from data, be it images or tabular data, allows processing of large amounts of data and increases temporal and spatial resolution.

In the case of brain tumors, these advancements also allow for the segmentation of intricate tissue architectures and the integration of multimodal data to improve diagnostic accuracy. One of the most pressing challenges in brain tumor research is understanding intra-tumoral heterogeneity (ITH), the dynamic and spatially variable nature of tumor populations that evolve rapidly and respond differently to treatment. Traditional biopsy-based molecular characterization tests are biased by sampling bias, missing critical regions of genetic diversity. Thus, creating a non-invasive method to characterize ITH across space and time is even more challenging. The Parker et al., study proposed a multimodal integration method of multiparametric magnetic resonance to map tumor properties across multiple spatial scales, from macro to micro and nano levels. The idea was to decode molecular and cellular behaviors from macro scales as provided by MRI. For this, incorporating genetic markers (e.g., IDH1 mutation, MGMT promoter methylation) with imaging features (e.g., signal intensity, diffusion metrics, perfusion parameters) was proposed, which provided a more accurate and dynamic measurement of tumor heterogeneity (76). The proposed model is a good example of the synergy between deep learning models; the authors propose to use convolutional neural networks for feature extraction from MRI images for tumor segmentation, while with generative adversarial networks, researchers can perform data augmentation, improve the quality of the images, and improve the generalization of the model. Beyond deep learning, a Random Forest was used to classify tumors based on molecular and imaging features. Support Vector Machine was used to identify the most informative imaging features linked to molecular subtypes. Although this study primarily serves as a roadmap review, the authors propose a pipeline that allows predictions to help classify treatment-resistant tumor subpopulations.

Additionally, drug perfusion in brain tumors is often very complex due to irregular vascularity and uneven passage through the BBB. Current methods, such as transcardial perfusion, cannot accurately determine drug extravasation because many remain in the vasculature, leading to potentially overestimated measurements. The study by Kostrikov et al., 3D deep tissue imaging with optical tissue clearing was used to capture three-dimensional images of the tumor vasculature. TRITC-dextran, a fluorescent marker that permeates tissues but not cells, was utilized to investigate extravasation. Further, the CNN model (VGG-19) was used for feature extraction or specific patterns in the tumor images while random forest was applied for classification particularly to identify extravasation points in the images. Moreover, to get better insight into the drug distribution within the tumor tissue, the researchers calculated the surface area and volume of the extravasation zones based on the number of identified spots and interestingly, found that the surface area and volume of extravasation zones were greater in more permeable areas of the tumor vasculature (77) (Table 4).

**Table 4:** Summary of methodological and computational development based studies for the management of brain disorders.

S.N.	Study	Objective	Target disease	Sample	Core method	Nanomaterial/Nanoscale	AI model
1.	Parker et al. (76)	Provide a “roadmap” for imaging genomics of brain tumors from multiparametric MRI signals, addressing intra-tumoral heterogeneity	Brain cancer	Brain tumor tissue	MRI scan	Nanoscale intra-tumoral heterogeneity (ITH)	Multiple (CNNs, GANs, SVMs, Random Forests, and AdaBoost)
2.	Kostrikov et al. (77)	Develop ML-based workflows for analyzing compound extravasation and tumor vasculature in large 3D cleared tissue datasets	Brain cancer	Brain tumor tissue	Optical imaging	Tramethylrhodamine (TRITC)-labeled dextran (hydrodynamic radius ~27 nm)	Deep Convolutional Neural network (VGG-19) and Random Forest
3.	Liu et al. (78)	Investigate nanoparticle–enzyme interactions (inhibition, binding) using a combinatorial gold nanoparticle library	Alzheimer’s disease	Protein–Nanoparticle complex	Quantitative Computational methods and molecular modeling	Combinatorial gold nanoparticles (Au NPs) library	Bayesian-Regularized Artificial Neural Network, and Multiple Linear Regression
4.	Zhang et al. (79)	Real-time detection of 20 amino acids using a copper (II)-functionalized MspA nanopore, enabling direct protein (or peptide) sequencing.	Parkinson’s disease	Pathological protein or peptide	Single Molecule Detection /Protein Sequencing	Copper (II)-functionalized MspA Nanopore	Random Forest, Naive Bayes, Neural Network, and k-NN

Apart from image-based analysis, molecular interactions are also studied at nanoscale resolutions. The study done by Liu et al., suggests that the catalytic activity of acetylcholinesterase (AChE) enzymes is inhibited by surface-functionalized gold nanoparticles (f-GNPs). This inhibition results in increased acetylcholine (ACh) release, which is responsible for neuroprotection against cholinergic neuron death in Alzheimer’s disease. In this study, a set of 47 f-GNPs was screened and experimental analysis showed that allyl-functionalized nanoparticles with fewer Hydroxyl group (-OH) showed maximum inhibition efficiency. Moreover, researchers trained a Bayesian neural network model to enhance the results and predict AChE inhibition by the f-GNPs. Thirteen molecular descriptors derived from quantitative structure–property relationship (QSPR) analysis were used as training data. Since nanoparticles can interact with AChE through various mechanisms including specific and nonspecific binding, lipophilic interactions, hydrogen bonding, and charge/dipole interactions these studies help identify the binding affinity and inhibition potential of different nanoparticle designs (78) (Table 4). Consequently, these results represent a significant advance in screening nanomaterials for treating cognitive disorders in Alzheimer’s disease, as they enable early prediction of nanoparticle efficacy and toxicity before synthesis. In the same line as the previous research, Zhang *et al.,* developed a copper (II)-functionalized *Mycobacterium smegmatis* porin A (MspA) nanopore to interact with proteins; they intended to generate a detection method of 20 proteinogenic amino acids. The authors used machine learning methods for the classifier, including Random Forest, Naïve Bayes, Neural Networks, and Ensemble methods as input data, they used blockade signals of amino acids passing through the nanopore and achieved a 99.1% classification accuracy with a 30.9% signal recovery rate. Also, this method is utilized for peptide sequencing associated with Alzheimer’s disease and cancer. Importantly, the sequencing of amyloid-beta (Aβ) peptides could be performed through this approach which is an important biomarker. Importantly, this new approach has detection limit in nanoscale which is less than 100 nM. It has potential to identified all amino acids, two post-translational modifications (PTMs), and one unnatural amino acid with 99.1% accuracy. Although this method is applicable for peptide sequencing but not for whole proteins sequencing. Thus, it offers a promising pathway toward nanopore-based proteomics (79) (Table 4).

## 4 Technical challenges, limitations and proposed solutions for AI driven nanomedicine in the management of brain disorders

The use of AI-based methods in nanomedicine has shown potential to advance various aspects of brain disorders management, including biomarker identification, diagnosis, prognosis and disease monitoring, drug delivery, therapeutics and computational and methodological advancements. Despite these developments and advancements, the included studies in this narrative review have some common technical challenges and limitations related to sample size, data sources, validation techniques, selection bias, methodological gaps, and overestimating of outcomes. One of the important limitations is the lack of large, high quality clinical datasets. Since the field of nanomedicine is in its development stage, the data requirements for complex AI-based learning and training models are not met (80, 81). For example, many studies relied on small sample sizes of patient cohorts (less than 100) or few animals or biological replicates (e.g., one mouse strain or 4–8 mice per group), that limit the statistical power and generalizability (Table 5). Additionally, some studies used simulated or synthetic data which lack the real-world biological variation (63). While few studies reported unusual deviation in sample size, e.g., Munteanu et al., used a large library of chemical drugs-nanoparticles pairs (855,129 drug-nanoparticle pairs) which, nonetheless, lacked diversity in chemical classes of drugs (71). Thus, while some studies were conducted on very small sample size (under 50 samples), they reported high diagnostic performance of AI based models, which raise overfitting concerns (46, 49). To address this, we must prioritize the development of high-throughput synthesis and characterization techniques, which is currently lacking. These platforms will enable the generation of the large, well-defined datasets like real biological datasets critical for advancing AI-driven nanomedicine research (82) (Table 5).

**Table 5:** Summary of major limitation categories and proposed solution across various extracted studies.

S.N.	Category	Key concern	Proposed solution
1.	Sample size	Underpowered studies, risk of overfitting and lack of external validation	Using multi-center cohorts or datasets, and pooled data from multiple origins
2.	Data sources	Single-center data, over-controlled setups, limited participant diversity, and questionable quality when merging data from multiple sources	Using multi-center cohorts or datasets, and including inclusive recruitment
3.	Validation techniques	Absence of external test sets, lack of benchmark comparisons, limited cross-validation, lack of hyperparameter tuning, lack of confidence intervals, and overstating performance	Using multi-center external test cohorts or datasets, using robust cross-validation (nested CV for tuning)
4.	Potential biases	Selection bias, model bias and lack of control for confounding variables	Stating explicitly the confounder variables, using multi-center cohorts or datasets
5.	Outcome limitations	Overgeneralization, missing clinical outcomes and lack of error analysis	Focusing on multiple performance metrics, using larger and more varied cohorts and datasets, including error analysis

Apart from this, other limitations are associated with data sources and validation techniques employed by the researchers to advance AI based nanomedicine. Most of the extracted studies are highly dependent on controlled laboratory or *in vitro* data, clinical datasets without cross-validation may limit the translational potential of AI-driven nanomedicines. Importantly, few studies showed noteworthy deviations because scientists relied upon on datasets derived from *in silico* or *in vitro* experiments (72, 78), while some studies utilized real human cohorts without multi-center validation (48, 58). The absence of external test sets, higher dependency on internal validation methods, and lack of comparison to baseline or alternative models were common in most of the extracted studies. For instance, one study utilized used 1,000 random splits but no independent dataset, while another study used diverse biophysical validation techniques but lacked clinical replication (46, 75). Additionally, selection biases are observed due to use of pre-selected features or homogeneous cohorts and model biases results because of idealized lab conditions or limited sensor variability. Also, most of studies do not consider demographic diversity, age, sex and comorbidities (56, 83) (Table 5).

Furthermore, other methodological gaps included limited model comparisons or hyperparameter tuning and lack of ablation studies, error analysis, or stratified performance metrics. For instance, one study used multimodal learning but did not benchmark against other ML models (64), and another report was solely based on ANN classifiers but had poor specificity in blinded tests (57). Moreover, some studies reported challenges regarding outcome-related limitations which are related to overstatement of performance metrics (e.g., 100% accuracy in small), outcomes being based on surrogate markers or non-clinical endpoints (e.g., fluorescence shift instead of patient diagnosis), and unevaluated outcomes if they remain reliable indicators over time (67, 69, 75) (Table 5). These limitations highlight the need for rigorous methodology and outcome assessment in AI-driven nanomedicine research for the management of brain related disorders.

Another key hurdle is the parametric optimization of complex nanostructures to produce effective nanotherapeutics. However, traditional methods struggle with this complexity which hinders the development of efficient nanostructures (84). To address this, AI based approaches such as machine learning algorithms are being employed. These methods enable the exploration and identification of optimal and effective nanostructure designs, ultimately leading to improved patient outcomes and reduced side effects (85, 86). Currently, health care professionals used AI-based nanorobot for controlled and targeted drug delivery for the managing brain disorders but, faces different challenges including precise control and maneuvering of nanorobots within the complex biological environment of the brain, as well as optimizing drug delivery across biological barriers (87).

To overcome these problems further advancements and implementations of complex ML based models are required. Additionally, another challenge is to understand increasing complexity of different AI based approach such as machine and deep learning model and how these models interpret data and make decisions to have safe and reliable applications in the development of advanced nanomedicine (88). Therefore, it is critical to understand how these AI models interpret the data and make the decisions to ensure safety and efficacy of these emerging technologies (89). Moreover, another problem is the translation of nanomedicine based *in vitro* and *in vivo* studies into effective clinical therapies. The major factors are toxicity, pharmacokinetics of nanomedicines as well as of their complex interactions with the immune systems (90). These problems can be addressed at some extent by applying machine learning, that offers some predictive capabilities, but the development of sophisticated AI methods is crucial for comprehensive analysis to ensure better safety and efficacy of nanomedicine (91). In conclusion, the future of brain disorder treatment strategies depends on the promising synergy between AI and nanomedicine. By overcoming various challenges and encouraging interdisciplinary collaboration, researchers and health professionals can develop innovative AI-driven nanomedicine that will revolutionize the different aspects of brain disorder management and improve the disease outcomes.

## 5 Conclusion and future prospects

The recent advancement in AI based nanomedicines leads to the improved management of brain disorders including brain cancer, Alzheimer’s disease, Parkinson’s disease, and multiple sclerosis. This innovative synergy improves disease diagnosis, biomarker identification, prognostic assessment and disease monitoring, targeted drug delivery, and therapeutic intervention as well as contributing to computational and methodological developments. One of the important advancements is being made in diagnosing brain disorders using AI-driven Nano diagnostics. The extracted studies demonstrated that these diagnostics have high accuracy, specificity and sensitivity in detecting Alzheimer’s, Parkinson’s, multiple sclerosis, and brain cancers through advanced biomarker analysis (SERS, EV detection, VOC analysis) and imaging techniques (MRI, nanoDSF). Further, prognosis is also improved, particularly ML/DL uses advanced imaging and single-molecule techniques to enable real-time disease monitoring by tracking oligomer aggregation, neurotransmitter fluctuations, and immune responses.

Additionally, other therapeutic advancements in brain disorder management utilize the computational models to identify effective drug-nanoparticle complexes, optimize drug delivery across the blood–brain barrier, and personalize neuromodulation through AI-enhanced deep brain stimulation. Furthermore, AI-based analysis of complex nanoscale interactions, improved image segmentation for tumor characterization, and facilitated molecular sequencing for peptide identification drive methodological advancements. Looking to the future, the continued advancements in the integration of AI driven methods in nanomedicine for brain disorders holds immense promise. However, researchers are focused on advancing the multimodal data integration, which will combine diverse data sources, including genomics, proteomics, and imaging to develop more comprehensive and predictive models. Furthermore, the development of explainable artificial intelligence (AI) is crucial for in-depth interpretability of AI models, thereby increasing clinical trust and facilitating translation. Additionally, personalized medicines which tailor’s treatments based on individual patient data and disease characteristics, is expected to improve therapeutic outcomes. Moreover, the current development in the field of nanopore-based sequencing and detection techniques advances the proteome-based diagnosis. In addition to this, scientists around the globe is looking for the development of robust models which can map and predicts the intra-tumoral heterogeneity (ITH) that enables the clinicians for providing effective treatment to brain cancer patients. Also, expanding data diversity by incorporating more diverse datasets from *in vivo* validation studies to train the AI model is essential to bridge the gap between *in vitro* and *in vivo* findings. In conclusion, the recent development of AI models and their integration with nanomedicine has advances different aspects brain disorders management and further, more research is needed to yield more innovative and transformative solutions for the management of brain related pathologies.
