# Supplementary material for: Artificial intelligence based advancements in nanomedicine for brain disorder management: an updated narrative review
Source: Front Med (Lausanne). 2025 May 13;12:1599340. doi: 10.3389/fmed.2025.1599340 (PMC12106332; doi:10.3389/fmed.2025.1599340)
Supplement: Supplementary file 1 [file Table_1.docx]

**Supplementary Materials**

**eTable 1:** Summary of Search Strategies and Retrieved Records

| Target Database | Summary of Search Strategy | Retrieved Articles |
| --- | --- | --- |
| PubMed | (nanomedicine[tiab] OR "nano medicine"[tiab] OR nanotechnolog*[tiab] OR "nanoscale medicine"[tiab] OR Nanomedicine [Mesh] OR "Nanotechnology"[Mesh] OR "photothermal theranostic"[tiab] OR "Theranostic Nanomedicine"[Mesh] OR nanoparticle*[tiab] OR nanomaterial*[tiab] OR nanostructure*[tiab] OR “nano particle*” [tiab] OR “nano material*” [tiab] OR “nano structure*” [tiab] OR "Nanostructures"[Mesh])  ("machine learning"[tiab] OR "deep learning"[tiab] OR "hierarchical learning"[tiab] OR "large language model*"[tiab] OR Deep Learning[Mesh] OR Machine Learning[Mesh] OR "reinforcement learning"[tiab:~2] OR "federated learning"[tiab] OR "neural network*"[tiab] OR "Large Language Models"[Mesh] OR Artificial Intelligence[Mesh] OR “artificial intelligence”[tiab] OR “machine prediction”[tiab] OR “predictive learning model*”[tiab] OR “sentiment analysis”[tiab] OR “natural language processing”[tiab] OR “machine pattern analysis”[tiab])  ("multiple sclerosis"[tiab] OR "Alzheimer s"[tiab] OR Alzheimer[tiab] OR Alzheimers[tiab] OR "Parkinson s disease"[tiab] OR "Parkinsons disease"[tiab] OR "Parkinson disease"[tiab] OR "brain cancer*"[tiab] OR "brain neoplasm*"[tiab] OR "brain tumor*"[tiab] OR "Parkinson Disease"[Mesh] OR "Alzheimer Disease"[Mesh] OR "Brain Neoplasms"[Mesh] OR "Multiple Sclerosis"[Mesh] OR "brain carcinoma*"[tiab] OR "brain malignan*"[tiab] OR "chariot disease"[tiab:~0] OR "disseminated sclerosis"[tiab] OR "insular sclerosis"[tiab] OR "sclerosis multiplex"[tiab] OR "idiopathic parkinsonism"[tiab] OR "Parkinson dementia complex"[tiab] OR "primary parkinsonism"[tiab])  English[lang] (2014:2024[pdat)  The final search query was created by combining all search terms #1, #2, #3, #4, and #5 with the 'AND' operator. | 43 |
| Scopus | Title-Abs-Key (nanomedicine OR "nano medicine" OR nanotechnolog* OR "nanoscale medicine” OR "photothermal theranostic" OR nanoparticle* OR nanomaterial* OR nanostructure* OR "nano particle" OR "nano material" OR "nano structure" OR "nano particles" OR "nano materials" OR "nano structures”)  Title-Abs-Key ("machine learning" OR “deep learning" OR "hierarchical learning" OR "large language model” OR "large language models" OR (reinforcement W/2 learning) OR "federated learning" OR "neural network" OR "neural networks" OR "artificial intelligence" OR "machine prediction" OR "predictive learning model" OR "predictive learning models" OR "sentiment analysis" OR "natural language processing" OR "machine pattern analysis")  Title-Abs-Key ("multiple sclerosis" OR "Alzheimer s" OR Alzheimer OR Alzheimers OR "Parkinson s disease" OR "Parkinsons disease" OR "Parkinson disease" OR "brain cancer" OR "brain neoplasm" OR "brain tumor" OR "brain cancers" OR "brain neoplasms" OR "brain tumors" OR "brain tumour" OR "brain tumours" OR "brain carcinoma" OR "brain malignancy" OR "brain carcinomas" OR "brain malignancies" OR "chariot disease" OR "disseminated sclerosis" OR "insular sclerosis" OR "sclerosis multiplex" OR "idiopathic parkinsonism" OR "Parkinson dementia complex" OR "primary parkinsonism")  (LIMIT-TO (LANGUAGE, “English”))  ( LIMIT-TO ( PUBYEAR , 2024 ) OR LIMIT-TO ( PUBYEAR ,2023 ) OR LIMIT-TO ( PUBYEAR , 2022 ) OR LIMIT-TO ( PUBYEAR , 2021 ) OR LIMIT-TO ( PUBYEAR , 2020 ) OR LIMIT-TO ( PUBYEAR , 2019 ) OR LIMIT-TO ( PUBYEAR , 2018 ) OR LIMIT-TO ( PUBYEAR , 2017 ) OR LIMIT-TO ( PUBYEAR , 2016 ) OR LIMIT-TO ( PUBYEAR , 2015 ) OR LIMIT-TO ( PUBYEAR , 2014 ) )  The final search query was created by combining all search terms #1, #2, #3, #4, and #5 with the 'AND' operator. | 166 |
| Embase | ('nanomedicine':ti,ab,kw OR 'nano medicine':ti,ab,kw OR 'nanotechnolog*':ti,ab,kw OR 'nanoscale medicine':ti,ab,kw OR 'nanomedicine'/exp OR 'nanotechnology'/exp OR 'photothermal theranostic':ti,ab,kw OR 'theranostic nanomedicine'/exp OR 'nanoparticle*':ti,ab,kw OR 'nanomaterial*':ti,ab,kw OR 'nanostructure*':ti,ab,kw OR 'nano particle*':ti,ab,kw OR 'nano material*':ti,ab,kw OR 'nano structure*':ti,ab,kw OR 'nanomaterial'/exp)  ('machine learning':ti,ab,kw OR 'deep learning':ti,ab,kw OR 'hierarchical learning':ti,ab,kw OR 'large language model*':ti,ab,kw OR 'deep learning'/exp OR 'machine learning'/exp OR ('reinforcement' NEAR/3 'learning'):ti,ab,kw OR 'federated learning':ti,ab,kw OR 'neural network*':ti,ab,kw OR 'large language model'/exp OR 'artificial intelligence'/exp OR 'artificial intelligence':ti,ab,kw OR 'machine prediction':ti,ab,kw OR 'predictive learning model*':ti,ab,kw OR 'sentiment analysis':ti,ab,kw OR 'natural language processing':ti,ab,kw OR 'machine pattern analysis':ti,ab,kw)  ('multiple sclerosis':ti,ab,kw OR 'alzheimer s':ti,ab,kw OR 'alzheimer':ti,ab,kw OR 'alzheimers':ti,ab,kw OR 'parkinson s disease':ti,ab,kw OR 'parkinsons disease':ti,ab,kw OR 'parkinson disease':ti,ab,kw OR 'brain cancer*':ti,ab,kw OR 'brain neoplasm*':ti,ab,kw OR 'brain tumor*':ti,ab,kw OR 'Parkinson disease'/exp OR 'Alzheimer disease'/exp OR 'brain tumor'/exp OR 'multiple sclerosis'/exp OR 'brain carcinoma*':ti,ab,kw OR 'brain malignan*':ti,ab,kw OR 'chariot disease':ti,ab,kw OR 'disseminated sclerosis':ti,ab,kw OR 'insular sclerosis':ti,ab,kw OR 'sclerosis multiplex':ti,ab,kw OR 'idiopathic parkinsonism':ti,ab,kw OR 'parkinson dementia complex':ti,ab,kw OR 'primary parkinsonism':ti,ab,kw)  [english]/lim [embase]/lim [2014-2024]/py  The final search query was created by combining all search terms #1, #2, #3, #4 #5 and #6 with the 'AND' operator. | 418 |
| Web of Science: Core Collection | TS= (nanomedicine OR "nano medicine" OR nanotechnolog* OR "nanoscale medicine” OR "photothermal theranostic" OR nanoparticle* OR nanomaterial* OR nanostructure* OR "nano particle" OR "nano material" OR "nano structure" OR "nano particles" OR "nano materials" OR "nano structures")  TS= ("machine learning" OR “deep learning" OR "hierarchical learning" OR "large language model” OR "large language models" OR (reinforcement W/2 learning) OR "federated learning" OR "neural network" OR "neural networks" OR "artificial intelligence" OR "machine prediction" OR "predictive learning model" OR "predictive learning models" OR "sentiment analysis" OR "natural language processing" OR "machine pattern analysis")  TS=("multiple sclerosis" OR "Alzheimer s" OR Alzheimer OR Alzheimers OR "Parkinson s disease" OR "Parkinsons disease" OR "Parkinson disease" OR "brain cancer" OR "brain neoplasm" OR "brain tumor" OR "brain cancers" OR "brain neoplasms" OR "brain tumors" OR "brain tumour" OR "brain tumours" OR "brain carcinoma" OR "brain malignancy" OR "brain carcinomas" OR "brain malignancies" OR "chariot disease" OR "disseminated sclerosis" OR "insular sclerosis" OR "sclerosis multiplex" OR "idiopathic parkinsonism" OR "Parkinson dementia complex" OR "primary parkinsonism")  (LA=("ENGLISH"))  PY= (2014-2024)  The final search query was created by combining all search terms #1, #2, #3, #4, and #5 with the 'AND' operator. | 60 |

**eTable 2:** Information collected for the 39 studies. The collected information was sectioned into different columns to identify the different applications of nanomedicine, ML, and DL and to categorize the studies into diagnostic, prognostic, therapeutic, and computational development.

| Covidence Number | Application | Study ID | Title | Aim of study | Study design | Target Disease | Machine learning/Deep learning | Type of Nanomedicine/Nanotechnology | Models used | Learning techniques | Classification/Prediction/Other | Metrics (values) | Cross-validation | Other information | Population description | Purpose of the study | Impact of the study |
| --- | --- | --- | --- | --- | --- | --- | --- | --- | --- | --- | --- | --- | --- | --- | --- | --- | --- |
| 80 | Diagnostic / Biomarker | Broza *et al,* 2017 | Exhaled Breath Markers for Nonimaging and Noninvasive Measures for Detection of Multiple Sclerosis | To develop a rapid, simple, non-invasive, and relatively  inexpensive point-of-care test approach for diagnosing  Multiple Sclerosis (MS). | Diagnostic test accuracy study | Multiple sclerosis | Machine Learning | To analyze sensor array data from nanomaterial-based breath analyzers Gas-chromatography linked with mass-spectrometry (GC-MS)  and nanomaterial-based sensors (termed, NA-NOSE). Sensor arrays | Multilayer perception type (MLP-type)  artificial neural networks (ANNs) | Supervised Learning | Classification: discriminate between individuals with multiple  sclerosis (MS) and healthy controls based on the analysis of  volatile organic compounds (VOCs) in their breath using  gas-chromatography mass-spectrometry (GC-MS) and a  nanomaterial-based sensor array 1. Blinded sets showed 95% positive predictive value (PPV) between MS- remission and control 2. 100% sensitivity with 100% negative predictive value (NPV) between MS not-treated (NT) and control.  3. 86% NPV between relapse and control. | 1. sensitivity, specificity, accuracy, positive predictive value (PPV), and negative predictive value (NPV) for both training and validation set. 2. blind set was categorized using ROC curve derived Youden's cutoff point. | 1. K-fold cross validation (K = 6) for evaluation of  the generalization capability and applicability span of the model  (2) different blind tests have been used. (MATLAB version 7.0.1.24704 (R14) is used for the designing and optimization of design of the MLPs.) | NA | Exhaled breath samples from 204 participants, including  146 multiple sclerosis (MS) patients and 58 healthy controls. Subgroups: remission (128 patients) and experiencing relapse (18 patients) Breath samples were collected at the Multiple Sclerosis Center,  Carmel Medical Center, Haifa, Israel gas chromatography-mass spectrometry (GC-MS) + nanomaterial-based  sensor array (NA-NOSE) -> to detect volatile organic compounds related to MS | Diagnostic | Nanotechnology-based sensors combined with an artificial neural network (ANN) for prediction models would be applicable for diagnosing or managing Multiple sclerosis conditions. |
| 109 | Diagnostic / Biomarker | Chung *et al,* 2021 | Plasma extracellular vesicles tau and ALPHA-amyloid as biomarkers of cognitive dysfunction of Parkinson's disease | To investigate the role of the plasma EV-borne tau and  alpha-amyloid as biomarkers for cognitive dysfunction in PD by investigating subjects with mild to moderate stage of PD  and non-PD controls | Case control study | Parkinson's disease | Machine Learning | To analyze plasma extracellular vesicles (EVs) containing tau and β-amyloid Type of Nano: Plasma Extracellular vesicles (EVs) -borne tau.  antibody functionalized magnetic nanoparticles | Artificial Neural Network | Supervised Learning | Classification: to identify cognitive dysfunction in Parkinson's  disease (PD) patients using plasma extracellular vesicle  (EV)-borne α-synuclein, tau, and β-amyloid 1-42 levels,  along with age and gender, as predicting factors | AUC of 0.911 for the validation set. | 4-fold cross-validation | The neural network structure is poor, and it is only mentioned that it was chosen empirically. With a logistic regression  they would have obtained the same. | Number of patients: 162.  Number of patients with PD: 116 Number of control group: 46 Ethnicity/race: NA Age: Control: 67.04 ± 7.04 PD: 69.66 ± 8.41 Female: Control: 28 PD: 54 MMSE: Control: 28.41 ± 1.24 PD: 24.17 ± 6 Data collection method in the study: plasma EVs were isolated,  and immunomagnetic reduction-based immunoassay was  used to assess the levels of alpha-synuclein, tau,  and ALPHA-amyloid 1-42 (beta1-42) within the EVs. | Biomarker Identification | Combining Artificial neural network (ANN) analysis with plasma EV biomarkers such as beta1-42 and tau, from patients enables  the precise identification of cognitive dysfunction in Parkinson's disease |
| 114 | Diagnostic / Biomarker | Corbo *et al,* 2021 | Analysis of the Human Plasma Proteome Using Multi-Nanoparticle Protein Corona for Detection of Alzheimer's Disease | To develop noninvasive diagnostics for early detection  of Alzheimer's Disease using multi-Nanoparticle  Protein Corona | Diagnostic test accuracy study | Alzheimer's disease | Machine Learning | ML was used to analyze proteomic changes and identify  disease-specific protein corona patterns. Type of Nano: 90–100 nm plain, amino-conjugated and  carboxyl-conjugated silica nanoparticles and 90–100nm plain,  amino-conjugated, and carboxyl-conjugated polystyrene nanoparticles | Random Forest Classifier | Supervised Learning | Classification: to develop a multi-nanoparticle protein corona  nanoplatform to distinguish between Alzheimer's disease (AD),  early-AD, and healthy individuals based on the protein corona  composition formed on the nanoparticles when incubated in plasma | AUC non-cohort samples: 99.8% AUC Cohort samples: 99.47% | 1000 random replications of training and testing splits | Feature importance is to identify the most important  protein-NP interactions | Plasma samples (11 AD samples and 8 healthy samples) were  randomly stratified into two sets: A training set of 10 samples  (6 AD and 4 healthy) and a test set of 9 samples (5 AD and 4 healthy) | Diagnostic | The noninvasive nano-platform, which converts protein changes  into disease identifiers, could enable early detection and  intervention for Alzheimer's and other diseases. |
| 142 | Diagnostic / Biomarker | Eid *et al,* 2024 | Machine learning-powered lead-free piezoelectric nanoparticle-based deep brain stimulation: A paradigm shifts in Parkinson's disease diagnosis and evaluation. | To develop a new machine learning (ML)optimized lead-free piezoelectric nanoparticle-based deep brain stimulation (LF-PND-DBS) system for diagnosing and evaluating Parkinson's disease. | Other: Computational/Experimental study | Parkinson's disease | Deep Learning | The DL method processes EEG images by identifying relevant  areas of neuronal activation associated with Parkinson's disease (attention maps?), thereby finding the optimal stimulation  parameters (frequency, amplitude, duration). Type of Nano: lead-free piezoelectric nanoparticles | Transformer networks, hybrid Simulated  Annealing-Particle Swarm Optimization (SA-PSO),  and Federated Learning (FL) | Supervised and Federate Learning | Classification: to classify Parkinson’s disease severity and  predicting the response to deep brain stimulation | Proposed model (TFA) Accuracy: 98.3 Precision: 98.2  Recall: 99.1 F1-Score: 99.2 sensitivity of 99.1% and a specificity of 98.2% | The model was trained on several epochs, additionally  Federated Learning was used, so it is likely to be  difficult to make use of cross-validation. | NA | Clinical and electroencephalography (EEG) data from Parkinson’s patients EEG dataset was sourced from Kaggle repositories | Diagnostic | Integration is a less invasive alternative to deep brain stimulation.  It also allows customization of treatment as the Transformer model  analyzes EEG to identify specific neural patterns in each patient.  This increases the accuracy of diagnosis and progression of the disease  by detecting early signs of Parkinson's disease. The integration allows for more precise and personalized stimulation, avoiding adverse effects, i.e., other areas are unaffected, which generates behavioral  or emotional changes. It incorporates an automatic and real-time  adjustment of the stimulation parameters (Simulated Annealing  and Particle Swarm Optimization). |
| 151 | Diagnostic / Biomarker | Etxebarria-Elezgarai *et al,* 2024 | Surface-Enhanced Raman Spectroscopy for Early Detection of Alzheimer's Disease | To develop an early detection method for Alzheimer's disease  by analyzing cerebrospinal fluid samples using surface-enhanced  Raman spectroscopy (SERS) with commercially available  gold nanoparticle substrates. | Diagnostic test accuracy study | Alzheimer's disease | Machine Learning | The ML method used the spectral data to classify PD or healthy  patients. The synergy between Nano and ML improves the  classification of AD patients in early stages. Type of Nano: plasmonic nanoparticles | partial least squares discriminant analysis | Supervised Learning | Classification: to distinguish between patients with Alzheimer's  disease (prodromal and preclinical stages) and healthy individuals  using Surface-Enhanced Raman Spectroscopy (SERS) of  cerebrospinal fluid (CSF) fractions and partial least squares discriminant analysis (PLS-DA) | 100% accuracy in classifying AD patients 85% for the healthy control group | They mentioned but they did not specified values | They selected 10 and 12 latent variables for the two  different CSF fractions | 51 samples were analyzed for the cerebrospinal  fluid-supernatant fraction (16 Prodromal stages,  15 Preclinical stages, 20 Healthy Individuals) and  45 samples for the cerebrospinal fluid-waste fraction  (10 Prodromal stages, 15 Preclinical stages, 20 Healthy Individuals) | Diagnostic; Biomarker Identification | This study proposes the integration of ML with nanotechnology methods (gold nanoparticles in SERS) to improve the early detection of diseases such as Alzheimer's. In addition, biomarkers of different molecular weights can be detected using cerebrospinal fluid. |
| 153 | Diagnostic / Biomarker | Eyraud *et al,* 2023 | Plasma nanoDSF Denaturation Profile at Baseline Is Predictive of Glioblastoma EGFR Status | To determine if nanoDSF-derived protein denaturation profiles (PDPs),  combined with artificial intelligence (AI), can accurately identify  EGFR alterations in glioblastoma (GBM) brain tumors | Cohort study | Brain Cancer | Machine Learning | ML models were trained to analyze plasma denaturation profiles  obtained via nanoDSF to distinguish molecular subtypes of GBM  (EGFR amplification) and assess post-surgical changes. Type of Nano: nonoDsf technology | Classical Logistic Regression (LR),  Support Vector Machine (SVM), and two Random Forest (RF) and Adaptive Boosting (AdaBoost). | Supervised Learning | Classification: classification of EGFR amplification and MGMT  promoter methylation in GBM using plasma denaturation profiles | Accuracy -> AdaBoost: 81.5% SVM: 55.6% RF: 63.0% LR: 59.3% | Leave-one-out cross-validation | They used as confounder factor surgery-induced inflammatory  response affected post-surgical PDPs. They did not perform hyperparameter tuning and model  optimization because the small dataset limited.  They used feature engineering by using the first derivatives  of fluorescence and scattering measures to capture dynamic changes | Local prospective cohort of 38 adult patients (≥18 years)  diagnosed with IDH wild-type glioblastoma (GBM) at Timone  Hospital (Marseille, France) between June 2016 and October 2017 Other variables: Age, gender, type of surgery, Karnofsky  Performance Status (KPS), oncological treatment,  clinical symptoms, steroid dosage, and MRI characteristics Molecular profiling was performed using Next-Generation  Sequencing (NGS) for EGFR amplification and pyrosequencing  for MGMT promoter methylation | Screening | GBM-specific baseline plasma PDP signatures offer potential for predicting the molecular profile of these tumors. |
| 304 | Diagnostic / Biomarker | Kim *et al,* 2024.1 | Distinct plasma phosphorylated-tau proteins profiling for the differential diagnosis of mild cognitive impairment and Alzheimer's disease by plasmonic asymmetric nanobridge-based biosensor | To develop a plasmonic asymmetric nanobridge (PAN) biosensor  for quantitative p-tau profiling in plasma, allowing for  the differentiation of MCI and AD and the determination of  its correlation with AD progression. | Diagnostic test accuracy study | Alzheimer's disease | Machine Learning | They used a SVM model to analyze and classify diagnostic data derived from the Plasmonic Asymmetric Nanobridge-based biosensor Type of Nano: Plasmonic asymmetric nanobridge (PAN)-based biosensor | Support vector machine (SVM) | Supervised Learning | Classification: to profile distinct plasma phosphorylated-tau  (p-tau) proteins using a plasmonic asymmetric nanobridge- based biosensor and support vector machine (SVM) classifier | 1. AUC (MCI patients Vs HCs) =0.9659,  AUC (MCI Vs AD patients) = 0.9063 2. Coefficient of determination R2 > 0.950 | Train-test split (60% training, 40% testing) | Quantitative profiling of p-tau expression levels (input feature) -> SVM model | Plasma samples from AD patients = 20 MCI patients = 16  Human controls (HCs) =11 | Diagnostic | The PAN-based plasmonic biosensor shows significant clinical potential for predicting asymptomatic Alzheimer's disease (AD) progression. |
| 297 | Diagnostic / Biomarker | Kim *et al,* 2024.2 | Surface-functionalized SERS platform for deep learning-assisted diagnosis of Alzheimer's disease | To establish a deep learning-assisted SERS platform  for separate blood amyloid beta(1-42) and metabolite  analysis, for Alzheimer's disease diagnosis | Diagnostic test accuracy study | Alzheimer's disease | Deep Learning | They used DL to distinguish spectral features of AD-related blood biomarkers from the interfering components in biofluids Type of Nano: Surface-functionalized SERS platform  ( Au nanowire arrays) | Feed-forward neural network | Supervised Learning | Classification: classification of SERS spectra into Alzheimer's  Disease vs. Healthy Control categories. Differentiation of  different stages of beta42 oligomerization in AD diagnosis | Oligomerization classification 96-100% accuracy Blood plasma classification,  sensitivity: 82.9%, and specificity: 92.2% | model trained on 10 epochs | ANN structure: Three hidden layers with sizes 2500, 1000,  and 100 neurons. ReLU activation function. | human blood plasma samples from two groups: 20 healthy controls (HC)  and 20 Alzheimer's disease (AD) patients HC -> Biobank of Chungnam National University Hospital AD -> Biobank of Pusan National University Hospital Plasma sample was analyzed using Surface-enhanced  Raman spectroscopy (SERS) | Diagnostic | This study demonstrates the potential of antibody immobilized and SAM-coated substrates, combined with deep learning, to revolutionize AD diagnosis through rapid and non-invasive blood  plasma analysis. |
| 333 | Diagnostic / Biomarker | Li *et al,* 2024 | Candidate biomarkers of EV-microRNA in detecting REM sleep behavior disorder and Parkinson's disease | To identify reliable, minimally invasive, plasma extracellular  vesicle (EV)-derived microRNA (miRNA) biomarkers for the  early detection and monitoring of Parkinson's disease (PD)  and idiopathic REM sleep behavior disorder (iRBD). | Diagnostic test accuracy study | Parkinson's disease | Machine Learning | They used ML models analyze EV-associated miRNA signatures  to differentiate between normal, iRBD, and PD patients Type of Nano: EV-microRNA | SVM | Supervised Learning | Classification: identification of diagnostic miRNA signatures for  distinguishing REM Sleep Behavior Disorder vs. Healthy individuals,  PD vs. Healthy individuals, PD vs. REM Sleep Behavior Disorder patients | iRBD vs. Healthy: AUC: 0.969 PD vs. Healthy: AUC: 0.916 PD vs. iRBD: AUC: 0.929 | 5-fold cross-validation | Selection of differentially expressed miRNAs They used EV-small sequencing for optimizing  library construction for small RNA inputs | No. of Participants: 169 (Three groups: 56 iRBD patients, 53 PD patients, and 60 healthy people) Age: 63.5 ± 9.0 (healthy) 64.0 ± 7.3 (iRBD) and 63.0 ±9.0 (PD) Sex(M/F): 32/25 (healthy), 34/22 (iRBD), 25/28 (PD). | Biomarker Identification | This study identifies plasma EV-derived miRNA biomarkers for the early detection and monitoring of Parkinson's disease (PD) and prodromal PD (iRBD). Also, highly accurate diagnostic signatures using optimized sequencing and machine learning, enabling differentiation between healthy, iRBD, and PD states and tracking iRBD conversion. This offers a minimally invasive tool for improved diagnosis, monitoring, and drug development. |
| 365 | Diagnostic / Biomarker | Meehan *et al,* 2024 | A reproducible approach for the use of aptamer libraries for the identification of Aptamarkers for brain amyloid deposition based on plasma analysis | To develop a blood-based diagnostic test for brain amyloid  deposition (a risk factor for Alzheimer's disease) using aptamer library | Diagnostic test accuracy study | Alzheimer's disease | Machine Learning | They used ML models to evaluate the predictive capability of  aptamer-based biomarkers (Aptamarkers) for detecting brain  amyloid deposition Type of Nano: Aptamer library | Extra Trees Classifier Random Forest Classifier Gradient Boosting Logistic Regression Decision Trees Support Vector Machines (SVM) Gaussian Naive Bayes Multi-Layer Perceptron (MLP) XGBoost Ridge Classifier | Supervised Learning | Classification: predicting high vs. low brain amyloid deposition  based on aptamer qPCR signals and clinical variables Identifying the most relevant aptamers for distinguishing  between amyloid-positive and amyloid-negative samples | Best Model (Extra Trees Classifier) -> AUROC: 0.79 Model including ApoE genetic status  (Random Forest) -> AUROC: 0.81 | 4-fold cross-validation | Features included normalized qPCR aptamer signals and  selected clinical variables Clinical variables considered: Age, sex, cognitive status | Plasma samples from 390 individuals obtained from  the Australian Imaging, Biomarkers, and Lifestyle (AIBL) study | Diagnostic | Aptamarkers advance disease diagnosis with non-invasive tests, exemplified by Alzheimer's detection, promising earlier intervention and improved outcomes. |
| 480 | Diagnostic / Biomarker | Rani *et al,* 2021 | Nanoscale imaging technique for accurate identification of brain tumor contour using the NBDS method | To develop a fast, accurate brain tumor detection and  classification system in MRI images using advanced image  processing, nanotechnology-based segmentation, and  deep neural networks. | Diagnostic test accuracy study | Brain Cancer | Deep Learning | The Nanotechnology-based detection scheme system enhances  segmentation accuracy by identifying tumor seed pixels at the  nanoscale. Deep learning classification is used for the final tumor categorization. Type of Nano: Nanotechnology-Based Detection Scheme (MATLAB).  The article talks about nanoscale detection. | Deep-neural network | Supervised Learning | Classification: the model classifies tumors into benign or  malignant categories using extracted features | Accuracy: 97.3% Sensitivity: 96.7% Specificity: 95.6% Precision: 98.4% | NA | Extracted features included mean, variance, skewness,  mutual information, and statistical measures of tumor shape and texture. | Kaggle Brain MRI Database -> 432 healthy brain images and 1,018 brain tumor images. The International Cancer Center Neyyoor (ICCN) ->  175 healthy brain images and 205 brain tumor images | Diagnostic | This study develops a fast, accurate (97.3%) AI system for brain tumor diagnosis from MRIs, enabling earlier detection and improved patient care through advanced image processing and nanotechnology-inspired segmentation. |
| 486 | Diagnostic / Biomarker | Resmi *et al,* 2024 | Ultrasensitive Detection of Blood-Based Alzheimer's Disease Biomarkers: A Comprehensive SERS-Immunoassay Platform Enhanced by Machine Learning | To introduce a SERS-based, machine-learning-driven method  for the ultrasensitive detection of multiple AD biomarkers | Diagnostic test accuracy study | Alzheimer's disease | Machine Learning | Machine learning was used to process and classify SERS spectral data,  distinguishing between different clinical groups (AD, MCI, and healthy controls) Type of Nano: Cs-AuNPs on Al Surface (SERS -immunoassay Platform) | Multilayer Perceptron (MLP) Radial Basis Function (RBF) Support Vector Machine (SVM) Linear Discriminant Analysis (LDA) | Supervised Learning | Classification: classification of control vs MCI, control vs AD, and MCI vs AD. Classification: differentiation of disease progression stages using spectral features. Classification: identifying significant biomarkers (beta42, beta40, p-tau, t-tau). | Linear Discriminant Analysis:  Sensitivity: 40-100% (Control vs. MCI),  46-100% (Control vs. AD), 40-88% (MCI vs. AD);  Specificity: 56-100% (Control vs. MCI),  60-100% (Control vs. AD), 63-88% (MCI vs. AD) | 70% training, 30% testing | For feature engineering:  Spectral data normalization PCA for dimensionality reduction Selection of spectroscopically significant bands | Blood plasma samples from 75 individuals recruited from  the Neurology Department of the Sree Chitra Tirunal Institute for Medical Sciences and Technology (SCTIMST). Three groups: 25 individuals diagnosed with Alzheimer's disease,  25 with mild cognitive impairment (MCI), and 25 healthy controls.  The control group consisted of age-matched volunteers  with no neurological or psychiatric disorders | Biomarker Identification | A novel SERS-based platform, combined with machine learning, enables ultrasensitive, non-invasive detection of AD biomarkers in blood, offering potential for early diagnosis and improved management. |
| 508 | Diagnostic / Biomarker | Ryzhikova *et al,* 2019 | Multivariate Statistical Analysis of Surface Enhanced Raman Spectra of Human Serum for Alzheimer's Disease Diagnosis | To develop a rapid, accurate, and minimally invasive blood test  for Alzheimer's disease (AD) diagnosis using surface-enhanced  Raman spectroscopy (SERS) coupled with multivariate statistical  analysis and artificial neural networks (ANNs) | Diagnostic test accuracy study | Alzheimer's disease | Machine Learning | DL models (ANNs) analyze SERS spectral data to differentiate  between AD, OD, and HC samples Type of Nano: Silver colloidal nanoparticle (AgNPs) | Artificial Neural Networks | Supervised Learning | Classification: Binary classification: Differentiating AD vs. HC.  Tertiary classification: Differentiating mild AD, moderate AD, and HC. Another tertiary model: Differentiating HC, AD, and OD. | Genetic Algorithm + Artificial Neural Networks: Binary Model (AD vs. HC): Accuracy: 96.47% Tertiary Model (Mild AD, Moderate AD, HC): Accuracy: 94.84% Tertiary Model (AD, HC, OD): Accuracy: 98.31% | Bootstrap Latin Partition (BLP) Cross-Validation | Genetic Algorithm (GA) was used for feature selection, optimizing spectral regions for classification. | Samples were obtained from neurological clinics at Albany Medical Center,  blood serum samples from a total of 48 individuals,  Three groups: Alzheimer’s disease patients (n = 20), patients with other  neurodegenerative dementias (OD) (n = 18), and healthy controls (HC) (n = 10).  The AD group included 10 mild and 10 moderate cases, while  the OD group comprised individuals diagnosed with Lewy body dementia (n = 5),  Parkinson’s disease dementia (n = 10), and frontotemporal dementia (FTD) (n = 3).  The dataset: 480 Raman spectra (10 spectra per sample) ->  analyzed using Surface Enhanced Raman Spectroscopy | Diagnostic | SERS-based blood test shows promise for rapid, accurate, and non-invasive Alzheimer's diagnosis, enabling earlier detection and improved patient care. |
| 569 | Diagnostic / Biomarker | Sun *et al,* 2024 | A radiometric SERS strategy for the prediction of cancer cell proportion and guidance of glioma surgical resection | To develop a rapid, label-free SERS strategy for intraoperative  glioma cell quantification | Diagnostic test accuracy study | Brain Cancer | Deep Learning | To analyze radiometric SERS signals from silver nanoparticles,  enabling precise quantification of glioma cell proportions for intraoperative tumor boundary detection. Type of Nano: Silver Nanoparticles (AgNPs) | Artificial Neural Network & Polynomial Regression Model | Supervised Learning | Regression: quantification of glioma cells using spectral peaks at 655 cm-1 and 717 cm-1. Classification: delineation of tumor margins during  intraoperative surgical resection. Classification: detection of residual tumor burden in frozen samples using AI models. | ANN Model: R2 = 0.83, RMSE = 0.26 Polynomial Regression: R2 = 0.85, RMSE = 0.19 | Internal validation was performed using 20% of the dataset  in the ANN model | Baseline correction, normalization, and feature selection was performed on Raman spectra. PCA and Partial Least Squares Discriminant Analysis (PLS-DA)  were used to separate glioma cells from normal brain tissue based on spectral patterns. | Simulated samples were prepared by homogenizing normal brain tissue and  patient-derived glioma cells (PDC-4, PDC-21, and PDC-63) in varying proportions. 31 frozen tumor samples from 20 glioma patients, obtained during surgical resections The dataset included RNA sequencing data | Diagnostic | This research developed a rapid, label-free SERS method for quantifying glioma cells during surgery. The method improves tumor margin detection and promises better surgical outcomes and broader cancer diagnostic applications. |
| 633 | Diagnostic / Biomarker | Wang *et al,* 2023 | Ultra-sensitive SERS detection of beta 1-42 for Alzheimer's disease using graphene oxide/gold nanohybrids | To develop a highly sensitive SERS method using GO/Au NPs  to detect beta1-42 for early Alzheimer's diagnosis | Diagnostic test accuracy study | Alzheimer's disease | Deep Learning | DL models were used to classify and quantify different fibrillation  stages of the Alzheimer's biomarker (beta1-42) based on  SERS spectral data Type of Nano: Graphene Oxide/ Gold Nanoparticles (GO/Au NPs) | Support Vector Machine (SVM) One-Dimensional Convolutional Neural Network  (1DCNN) (based on ResNet) | Supervised Learning | Classification of different fibril states of beta1-42 (0-120 hours of incubation). | SVM Accuracy: 88.33% 1DCNN Accuracy: 98.9% | 1DCNN optimized with Adam optimizer and cross-entropy loss function | They extracted Raman spectral features (e.g., peak intensities) as input for classification. | Solutions of Aβ 1–42 (ranging from 0.1 to 1 ng/mL)  obtained from Sigma-Aldrich + fetal bovine serum (FBS) | Diagnostic | This study introduces a highly sensitive SERS method using GO/Au nanohybrids to detect low levels of beta1-42, enabling earlier Alzheimer's diagnosis. This method offers improved sensitivity for AD biomarker detection, broader neurodegenerative disease research potential, and the possibility of developing portable, noninvasive diagnostic tools. |
| 636 | Diagnostic / Biomarker | Wang *et al,* 2024.1 | Integrated Ultrasound-Enrichment and Machine Learning in Colorimetric Lateral Flow Assay for Accurate and Sensitive Clinical Alzheimer's Biomarker Diagnosis | To develop a machine learning-optimized lateral flow assay (LFA) with ultrasound enrichment for highly sensitive and accurate  detection of tau proteins in Alzheimer's disease diagnosis. | Diagnostic test accuracy study | Alzheimer's disease | Machine Learning | Integration of ultrasound-enriched colloidal gold nanoparticles  (AuNPs) in a lateral flow assay (LFA) type of Nano: colloidal gold nanoparticle | K-nearest neighbor (KNN) and  Gaussian process regression (GPR) | Supervised and Unsupervised Learning | Classification: k-nearest neighbor (KNN) for classification of  samples based on distance in concentration classes.  Regression: Gaussian process regression (GPR) for  quantification (regression) of low abundance detectors | KNN algorithm as a classifier and the GPR algorithm as a  quantifier, achieving 98.11% accuracy in differentiation  and 99.99% accuracy in quantification against undiluted samples. | 5-fold cross-validation | NA | Clinical samples were obtained from  Longgang District Central Hospital of Shenzhen.  Six clinical plasma samples for validation. | Biomarker Identification | ML and Nano leverage ultrasound-enriched colloidal gold nanoparticles to enhance the signal. KNN and GPR process and quantify colorimetric data, resulting in highly sensitive and accurate tau protein detection for Alzheimer's diagnosis. |
| 629 | Diagnostic / Biomarker | Wang *et al,* 2024.2 | A distinction of gliomas at cellular and tissue level by surface-enhanced Raman scattering spectroscopy | To develop SERS-based methods, combined with machine learning,  for rapid and accurate detection and characterization of glioblastoma  multiforme (GBM) at both cellular and tissue levels, including tumor  grading and IDH mutation identification, for potential  intraoperative diagnosis. | Other: Methodological Development | Brain Cancer | Machine Learning | Machine learning algorithms were used to analyze and classify  SERS spectral data to differentiate glioma from trauma tissues  and to distinguish tumor grades and IDH mutations Type of Nano: Gold nanoshell (SiO2@Au) & Gold nanoisland (AuNI) | Support Vector Machine (SVM) with a linear kernel. Orthogonal Partial Least  Squares Discriminant Analysis (OPLS-DA). | Supervised Learning | Classification: Differentiation of glioma cells from normal  astrocytes and non-CNS tumor cells. Classification of  glioma vs. trauma tissues. Distinguishing tumor grades and  identifying IDH mutations. | OPLS-DA model: sensitivity of 100%, specificity of 87.50%,  and an overall accuracy of 97.50%.  ROC AUC Values: Over 0.99 for all classification tasks. | 7-fold Cross-Validation | SERS spectral data were preprocessed, and  specific Raman peak intensities were used as features. | SERS spectra from tissue samples of 64 glioma patients and  16 brain trauma patients Four different cell types: normal human astrocytes (HA1800),  human glioma cell lines (U87 and U251), and a human myeloma cell line (U266) | Diagnostic | This study demonstrates that SERS combined with machine learning offers rapid, accurate glioma detection and characterization at cellular and tissue levels, enabling improved diagnostics, surgical guidance, and treatment decisions. |
| 645 | Diagnostic / Biomarker | Xu *et al,* 2022 | Machine Learning-Assisted Sensor Array Based on Poly(amidoamine) (PAMAM) Dendrimers for Diagnosing Alzheimer's Disease | To develop a fluorescent sensor array composed of three  modified polyamidoamine dendrimers | Diagnostic test accuracy study | Alzheimer's disease | Machine Learning | They used ML models to process fluorescence pattern data from the sensor array, allowing classification of different  aggregation states of beta40/beta42 proteins Type of Nano: Fluorescent sensor (pyrenemodified G5  PAMAM dendrimers) | Linear Discriminant Analysis (LDA) Decision Tree (DT) Support Vector Machine (SVM) Logistic Regression (LR) | Supervised Learning | Classification: differentiation of beta40 and beta42 aggregates in different structural states (monomer, oligomer, fibril). Identification of beta biomarkers in various biological fluids, including serum and cerebrospinal fluid. Regression: Testing of the sensor array's performance under interference from metal ions and other proteins. | LDA classification accuracy: 100% for protein discrimination. Prediction accuracy in serum samples: 88.9%. AUROC for distinguishing beta40 and beta42 in cerebrospinal fluid: 96.6% | leave-one-out cross-validation | For feature engineering, fluorescence intensity changes  at different pH levels (5.0, 7.4, 9.0) were used to optimize  signal differentiation | Amyloid-β (Aβ) protein aggregates (Aβ40 and Aβ42) in  different aggregation states (monomer, oligomer, and fibril).  Also, real-world data -> serum and cerebrospinal fluid (CSF) | Diagnostic | This study introduces a highly accurate fluorescent sensor array, demonstrating 100% accuracy in general protein discrimination and detection of Alzheimer's disease biomarkers, beta40 and beta42 aggregates, even within complex biological samples. |
| 653 | Diagnostic / Biomarker | Xu *et al,* 2023.1 | Machine Learning-Assisted Nanoenzyme/Bioenzyme Dual-Coupled Array for Rapid Detection of Amyloids | To develop a fluorescent sensor array integrating AuNPs and  horseradish peroxidase for amyloid detection | Diagnostic test accuracy study | Alzheimer's disease | Machine Learning | They used ML models to optimize sensor element selection,  reducing redundant features while maintaining high  prediction accuracy. Type of Nano: Fluorescent Sensor (Gold Nanoparticles (AuNPs) & Bioenzyme (Horseradish Peroxidase) | Linear Discriminant Analysis (LDA) K-Nearest Neighbors (KNN) | Supervised Learning | Classification: to classify different amyloid species, including  monomers, oligomers, and fibrils, which correspond to  different Alzheimer's disease stages | k-nearest neighbors classifier, best model: 100% accuracy  in classifying amyloid-beta aggregation states.  AUROC between AD vs. Healthy Mice: 1 | 10-fold cross-validation | Feature selection was performed using the K-best model with ANOVA F-values | Synthetic amyloid peptide samples and blood plasma samples  'from AD model mice and healthy mice | Diagnostic | Developed a highly sensitive nanoenzyme-amplified sensor array, coupled with machine learning, for accurate amyloid-beta (beta) peptide discrimination. It enables ultralow detection and differentiation of beta aggregates, offering potential for improved Alzheimer's diagnosis and large-scale biomolecule screening. |
| 650 | Diagnostic / Biomarker | Xu *et al,* 2023.2 | Diagnosis of Parkinson's Disease via the Metabolic Fingerprint in Saliva by Deep Learning | To develop a noninvasive, high-throughput, and highly  reproducible diagnostic platform for Parkinson's disease  using saliva metabolic fingerprinting. | Diagnostic test accuracy study | Parkinson's disease | Machine Learning / Deep Learning | They integrated nanoparticle-enhanced LDI-MS for high-throughput  saliva metabolic fingerprinting with deep learning model Type of Nano: ferric particles | Least Absolute Shrinkage and Selection Operator XGBoost Support Vector Machines Random Forest Adaptive Boosting Stroke Network | Supervised Learning | Classification: Parkinson's Disease (PD) patients and healthy controls | Stroke Network (SN), best model: training cohort, AUC of 0.8009, sensitivity of 81.01%, and specificity of 80.18%.  validation cohort, AUC of 0.8496, sensitivity of 88.01%, and specificity of 79.18% | 10-fold-CV | NA | 312 participants: 187 Parkinson's Disease (PD) patients,  125 Healthy Controls (HC) | Diagnostic; Biomarker Identification | Combining both methodologies allows an automated, scalable, and high-accuracy metabolic data interpretation method. On the other hand, it is noninvasive and early detection, which improves the accuracy of detection, as it can be used for detecting PD in asymptomatic or early-stage patients. It allowed the identification of biomarkers; for example, the study highlighted the molecules 7-hydroxyprogesterone, creatinine, creatine, and ADP. |
| 663 | Diagnostic / Biomarker | Yu *et al,* 2022 | The Feasibility of Early Alzheimer's Disease Diagnosis Using a Neural Network Hybrid Platform | To develop a high-sensitivity and high-specificity diagnostic  tool by detecting biochemical changes in cerebrospinal fluid (CSF)  associated with AD using a neural network hybrid platform that  combines surface-enhanced Raman spectroscopy (SERS) and  convolutional neural networks (CNNs) | Diagnostic test accuracy study | Alzheimer's disease | Deep Learning | They used deep learning to interpret nanoparticle-enhanced  mass spectrometry for disease biomarker discovery Type of Nano: nanopores (specifically α-hemolysin  nanopores and the engineered MspA-N91H nanopore) | 1D-Convolutional Neural Network | Supervised Learning | Classification: hierarchical cluster analysis (HCA) to group  samples based on their SERS spectra, achieving concordance with sample provenance, and to distinguish between clinically evident AD and pre-clinical AD (FAD samples) | overall accuracy of 92%, with 100% accuracy for normal individuals and 88.9% accuracy for AD patients | leave-one-group-out cross-validation | Structure of the CNN: Convolutional Layers Max-Pooling Layers Fully Connected (Dense) Layers  They used data augmentation Random shifting of spectra by 1-2 wavenumbers. Addition of random noise. Generation of linear combinations of spectra from the same sample. | 30 cerebrospinal fluid (CSF) samples were obtained from patients at the University of California, Irvine (UCI)  Institute for Memory Impairment and Neurological Disorders (ADRC). | Diagnostic; Biomarker Identification | This study delves into improving the early and accurate detection of Alzheimer's disease. It achieved much better detection than conventional methods. It has become a non-invasive  and cost-effective diagnostic test. It reduces human intervention in interpreting the results of SERS spectra. |
| 312 | Methodological / Computational Development | Kostrikov *et al,* 2021 | Optical tissue clearing and machine learning can precisely characterize extravasation and blood vessel architecture in brain tumors | To develop two machine learning-based workflows for  semi-automated image analysis, enabling detailed quantification  of compound extravasation and tumor angioarchitecture in large  3D cleared tissue datasets | Other: Methodological Development | Brain Cancer | Deep Learning | They used ML to segment and analyze tumor vasculature and  compound extravasation patterns Type of Nano: Tramethylrhodamine (TRITC)-labeled dextran  (hydrodynamic radius ~27 nm) | Deep convolutional neural network (VGG-19) and random forest | Supervised Learning | Classification: the model classified and segmented different  vascular structures, categorized regions based on the degree  of transcardial perfusion, and identified extravasation spots of  injected compounds | They compared human annotators vs model, the metrics are related to the accuracy and  precision of extravasation and vasculature analysis. Machine learning workflow provided larger  spot counts compared to manual annotation. | They compared model's segmentation results with manual annotations | Blind deconvolution to reduce out-of-focus light and  improve segmentation accuracy | Orthotopic xenograft models of glioblastoma (GBM) in NMRI-nude mice syngeneic colorectal cancer models in BALB/c and C57BL/6 mice | Other: Methodological development | The methodology enables precise and comprehensive analysis of extravasation in brain tumors, facilitating the correlation of extravasation patterns with specific features of the heterogeneous tumor vasculature |
| 339 | Methodological / Computational Development | Liu *et al,* 2015 | Probing enzyme -nanoparticle interactions using combinatorial gold nanoparticle libraries | To elucidate the complex relationships between nanoparticle  surface chemistry and their binding interactions with proteins,  specifically acetylcholinesterase (AChE), using a combinatorial approach. | Other: Methodological Development | Alzheimer's disease | Machine Learning | The ML models predicted the nanoparticle-enzyme interactions, helping to identify specific nanoparticle surface chemistries  that strongly influence AChE inhibition and binding Type of Nano: Gold nanoparticle | Bayesian Regularized  Artificial Neural Network Multiple Linear Regression  with Expectation Maximization | Supervised Learning | Regression: prediction of AChE inhibition levels based on  nanoparticle surface chemistry | Linear Model: r2 (train) = 0.91, r2 (test) = 0.81 Bayesian Neural Network: r2 (train) = 0.87, r2 (test) = 0.81 | Train-Test Split (80% training, 20% testing) | Several features were extracted from the nanoparticle surface chemistry: Spatial moments of electronegativities (DISPe) Molecular shape indices (G2v, WHIM descriptors) Radial distribution functions (RDF140m) Autocorrelation functions (R3m+, R2u+) Lipophilicity (LogP) Functional group counts (e.g., hydroxyl, carboxyl groups) | A combinatorial library of 47 surface-modified gold nanoparticles (f-GNPs) It contains measurements of AChE binding, enzyme inhibition,  and fluorescence quenching, collected through Western blot analysis,  enzymatic activity assays, and fluorescence spectroscopy | Other: Methodological development | Study provides a more detailed understanding of the complex relationships between nanoparticle surface chemistry and protein binding, specifically with acetylcholinesterase (AChE). Also, the models created can be used to predict potentially harmful interactions, therefore advancing nanotoxicology studies. |
| 443 | Methodological / Computational Development | Parker *et al,* 2023 | Targeting intra-tumoral heterogeneity of human brain tumors with in vivo imaging: A roadmap for imaging genomics from multiparametric MR signals | To develop in vivo brain tumor cellular and molecular mapping  for optimized cancer treatment strategies | Other: Roadmap Review Study | Brain Cancer | Machine Learning / Deep Learning | They used ML models to enhance MRI capabilities by mapping cellular and molecular tumor features at a microscopic scale Type of Nano: Nanoscale Intra-Tumoral Heterogeneity (ITH) | Generalized Additive Models (GAM) Convolutional Neural Networks (CNNs) Generative Adversarial Networks (GANs) Adaptive Boosting (AdaBoost) Support Vector Machines (SVMs) Random Forests | Supervised, Unsupervised and  Semi-supervised Learning | Classification: classification of tumor heterogeneity based on MR imaging. Regression: prediction of tumor recurrence risk and treatment response. Classification: segmentation of tumor regions in MRI scans. Classification: estimation of cellular and molecular properties from MRI features. | RMSE for predicting cell density: 1.06 × 10³ cells/mm² (GAM model). Voxel-wise classification accuracy (CNN): >95% | NA | NA | Multiparametric MRI (mpMR) imaging Genomic and histopathological information | Other: Methodological Development | 1. This roadmap could significantly advance brain tumor research and clinical management, leading to more effective and personalized treatments. 2. It sets a framework for future research that integrates cutting-edge technologies to address the challenges of tumor heterogeneity and treatment resistance. |
| 677 | Methodological / Computational Development | Zhang *et al,* 2024 | Real-time detection of 20 amino acids and discrimination of pathologically relevant peptides with functionalized nanopore | To build a copper(II)-functionalized Mycobacterium smegmatis  porin A (MspA) nanopore with the N91H substitution, which enables direct identification of all 20 proteinogenic amino  acids when combined with a machine-learning algorithm. | Other: Computational/Experimental study | Parkinson's disease | Machine Learning | After detecting the amino acid signals, ML is used to classify  an amino acid. This opens the possibility of sequencing  proteins without the need for mass spectrometry. Type of Nano: nanopores (specifically α-hemolysin  nanopores and the engineered MspA-N91H nanopore) | Random Forest Naive Bayes Neural Network k-Nearest Neighbors Bagged CART AdaBoost | Supervised Learning | Classification: to identify amino acids based on signals  obtained from nanopore translocation events | Random Forest: 0.996 (training), 0.993 (testing), and 0.989 (validation) Accuracy of the model reached 99.1% when using 30.9% of the signals Limit of Detection (LOD) for Glycine was <100 nM | 10-fold-CV |  | The data sample consists of electrophysiology recordings from  experiments that detect and classify 20 proteinogenic amino acids,  two post-translationally modified (PTM) amino acids, and  one unnatural amino acid | Other: Molecular sequencing | The biomedical applications are interesting in the sense that it can sequence the 20 proteinogenic amino acids and their post-translational modifications. No replication or amplification  of the sequences is required to obtain a good signal. It allows the identification of proteoforms; for example, alpha-amyloid  mutant peptides were differentiated. The incorporation of ML  increased the accuracy of detection. |
| 95 | Monitoring / Prognosis | Chan *et al,* 2022 | Monitoring Amyloidogenesis with a 3D Deep-Learning-Guided Biolaser Imaging Array | To develop a peptide-encapsulated droplet microlaser  to monitor the amyloidogenesis process and evaluate  the efficacy of anti-amyloid drugs. | Diagnostic test accuracy study | Alzheimer's disease; Parkinson's disease | Deep Learning | Detection of amyloid peptide and nanostructure conformation  using protein-based microdroplet laser array. Type of Nano: two PAH (Polycyclic Aromatic Hydrocarbon) sensors | Multimodal learning (MML) comprised of  five 2D convolutional layers,  five 1D convolutional layers, and two fully connected layers | Supervised Learning | Classification: in this study a 3D deep-learning strategy is  developed to classify laser images from peptide-encapsulated  droplet microlasers to monitor the progression of  the amyloidogenesis process and evaluate the efficacy of anti-amyloid drugs | Confusion Metrics for all data sets including training,  validation and Test set (accuracy for all dataset is over 95%) | k-fold cross-validation (k=10) was used to test the CNN model | NA | Laser emission images from microdroplets containing native insulin. The dataset included images collected at different time intervals to monitor protein aggregation stages | Screening; Diagnostic | The integration of these biosensors with deep learning offers high-throughput drug screening platforms that may help scientist to develop therapeutics and diagnostic devices for neurodegenerative diseases, such as Alzheimer's and Parkinson's. |
| 118 | Monitoring / Prognosis | Crimi *et al,* 2014 | Predictive Value of Imaging Markers at Multiple Sclerosis Disease Onset Based on Gadolinium- and USPIO-Enhanced MRI and Machine Learning | Characterize patients with clinically Isolated Syndrome  from spatio-temporal lesion imaging to improve prognosis  and better treatment in the early stages of multiple sclerosis. | Non-randomized experimental study | Multiple sclerosis | Machine Learning | To classify spatio-temporal lesion patterns in Multiple Sclerosis  patients using USPIO-enhanced MRI (a nanotechnology-based  contrast agent that highlights macrophage activity) Type of Nano: Ultrasmall Super Paramagnetic Iron Oxide (USPIO)  and Gadolinium | Regression Model | Supervised Learning | Classification: to identify a pattern of multiple sclerosis lesions  using contrast agents and spectral clustering. Regression:  to relate the volume of chronic hypointense lesions to  patient classification | R2 score of 0:90. | NA | NA | 25 Clinically Isolated Syndrome (CIS) patients  (17 women and 8 men) aged 32.9 ± 8.6 years, recruited  from multiple French centers between July 2009 and April 2011 | Other: Early Prognosis | This approach offers a potential refinement of MS lesion classification beyond traditional measures, which helps to improve patient stratification for clinical trials focused on preventing or delaying disease progression and disability. |
| 227 | Monitoring / Prognosis | Hozhabr *et al,* 2024 | Machine Learning-Empowered Multicolor Detection and Discrimination of Dopaminergic Agents: Utilizing Gold Nanorods for Generating Rainbow Signals | To create an improved method for rapid and sensitive multianalyte  detection of dopaminergic agents to accurately assess  Parkinson's disease progression. | Diagnostic test accuracy study | Parkinson's disease | Machine Learning | Machine learning helped analyze spectral data and differentiate between L-DOPA,  Carbidopa, and Benserazide by recognizing color variations and plasmonic shifts  due to chemical interactions. Type of nano: Gold nanorods | Linear discriminant analysis (LDA) for pattern recognition and partial least-squares regression (PLSR) for regression analysis | Supervised Learning | Classification: to discriminate between different dopaminergic  agents (L-DOPA, Carbi, and Benz) and their mixtures using a  multicolorimetric sensor based on the inhibition etching of  gold nanorods (AuNRs) and linear discriminant analysis (LDA).  Regression: quantitative assessment of individual analytes and  their mixtures using partial least squares regression (PLSR) | L-DOPA: R2 = 0.9993, Limit of Detection = 0.9 μmol/L Carbidopa: R2 = 0.9702, Limit of Detection = 0.9 μmol/L Benserazide: R2 = 0.9767, Limit of Detection = 0.4 μmol/L Accuracy = 1, Sensitivity = 1, and Selectivity = 1,  for all classes (L-DOPA, Carbi, Benz, and their binary and  ternary mixtures) | Leave-One-Out Cross-Validation for LDA Venetian Blind Cross-Validation and test-set validation for PLSR | For feature Engineering, they used PCA for LDA. | Spectroscopic and colorimetric responses of gold nanorods (AuNRs)  subjected to etching inhibition by dopaminergic agents—levodopa (L-DOPA),  carbidopa (Carbi), and benserazide (Benz). The dataset includes UV–vis absorption spectra (350–950 nm)  and corresponding color variations real sample analysis using pharmaceutical tablets (Norstor and Parkin-C Fort) | Diagnostic | This study allows for the rapid and accurate detection of dopaminergic agents using machine learning-assisted, color-based sensors using etched gold nanorods. Also, it performed excellently in pharmaceutical quality control and demonstrates potential for portable, on-site use. |
| 296 | Monitoring / Prognosis | Kim *et al,* 2021 | Stretchable and self-healable catechol-chitosan-diatom hydrogel for triboelectric generator and self-powered tremor sensor targeting at Parkinson disease | To develop a stretchable, ionically conductive CCDHG and  a self-powered tremor sensor for Parkinson's disease monitoring | Other: Computational/Experimental study | Parkinson's disease | Machine Learning | A 3D AuNW substrate fabricated from gold nanowire arrays for SERS-based biomarker analysis Type of Nano: stretchable and self-healable catechol-chitosan-diatom  hydrogel (CCDHG)-based triboelectric nanogenerator (TENG) | Support Vector Machine (SVM) and K-Nearest Neighbor (KNN) | Supervised Learning | Classification: to classify Parkinson’s disease tremor severity  using a machine learning model based on voltage signals  from a self-powered tremor sensor | Accuracy achieved by the linear SVM model was 100% | 5-fold cross-validation | For feature engineering, they extracted: maximum peak frequency, root mean square of peak frequencies, maximum power spectral density, and integral of power spectral density | Fabrication and testing of the CCDHG using biomaterials such  as catechol, chitosan, and diatom frustules, followed by mechanical,  electrical, and adhesion strength evaluations -> detects low-frequency vibrations | Other: Monitoring Disease Progression | The study pioneered catechol-chitosan-diatom hydrogel (CCDHG) as a stretchable electrode in triboelectric nanogenerators (TENGs). The CCDHG's mussel-inspired catechol chemistry enabled strong adhesion to hydrophobic polymers, and the resulting tremor sensor, analyzed with machine learning, effectively identified Parkinson's disease patient conditions. |
| 309 | Monitoring / Prognosis | Komoto *et al,* 2020 | Time-resolved detection of neurotransmitters in mouse brain tissue for PD diagnostics | To develop a method for high temporal resolution mapping  of neurotransmitter distribution in the brain | Diagnostic test accuracy study | Parkinson's disease | Machine Learning | They used ML to enhance the discrimination of neurotransmitter  signals by analyzing current waveforms Type of Nano: Nanogap electrodes | XGBoost classifier Random Forest classifier | Supervised Learning | Classification: to classify dopamine (DA), serotonin (5-HT),  and norepinephrine (NE) from single-molecule conductance signals | F1-score = 0.52 vs random classification F1-score of 0.33 Accuracy of classification improved with  signal accumulation -> 80% for 20 signals, 90%  for 40 signals, and 99% for 110 signals | Tenfold cross-validation | Noise signals from electrode migration and non-target  molecules were removed. | Dopamine (DA), serotonin (5-HT), and norepinephrine (NE) in aqueous solutions -> mouse brain tissue samples. Brain tissue was obtained from a 10-week-old female C57BL/6J mouse -> mounted on a mechanically controllable break junction (MCBJ)  substrate for electrical measurements | Diagnostic | This study provides a powerful new tool for investigating neurotransmitter dynamics in the brain, which might help develop new diagnostic tools and therapeutic strategies for neurological disease. |
| 386 | Monitoring / Prognosis | Morris *et al,* 2020 | Engineered immunological niches to monitor disease activity and treatment efficacy in relapsing multiple sclerosis | To develop and validate a novel, minimally invasive,  biomaterial-based immunological niche system in a  mouse model of multiple sclerosis (MS) to serve as a  platform for real-time monitoring of immune activity  within target tissues. | Diagnostic test accuracy study | Multiple sclerosis | Machine Learning | They used ML models to analyze the gene expression changes  induced by nanoparticle treatments, distinguishing effective  from ineffective therapies and monitoring disease progression. Type of Nano: Antigen encapsulating PLG nanoparticles | Singular Value Decomposition Bootstrap Aggregated  Decision Tree Ensemble | Supervised and Unsupervised Learning | Classification: classification of healthy vs. diseased mice based  on gene expression data. Prediction of disease onset and  severity using gene signature scores. | AUC: 0.97-1 (95% CI: 0.89-1.06) | Leave-One-Out Cross-Validation | Gene selection was optimized using elastic net regularization to extract the most relevant predictors of disease state | Mouse model of multiple sclerosis (experimental  autoimmune encephalomyelitis (EAE) model). gene expression profiles from subcutaneous INs implanted in SJL/J mice immune cell populations in the INs, blood,  and spleen -> assessed via flow cytometry | Diagnostic; Other: Prognosis | Engineered niche technology enables early MS relapse detection, guides targeted therapy, reveals hidden immune dysfunction, and improves autoimmune disease management. |
| 395 | Monitoring / Prognosis | Muraoka *et al,* 2021 | Proteomic Profiling of Extracellular Vesicles Separated from Plasma of Former National Football League Players at Risk for Chronic Traumatic Encephalopathy | To investigate the potential of plasma extracellular vesicle (EV)  protein profiles as diagnostic biomarkers for Chronic Traumatic Encephalopathy (CTE) in former National Football League (NFL) players | Diagnostic test accuracy study | Alzheimer's disease | Machine Learning | They used ML models to analyze proteomic profiles of Plasma  Extracellular vesicles (Evs) and identify potential biomarkers  for Chronic Traumatic Encephalopathy Type of Nano: Size-exclusion chromatography -> separate Evs and nanoparticle tracking analysis -> characterize Evs | Linear Discriminant Analysis (LDA) Naive Bayes Support Vector Machine (SVM) | Supervised Learning | Classification: machine learning model classified former  National Football League players at risk of CTE versus control  individuals based on proteomic signatures in EVs | The ensemble classifier achieved 85% accuracy. AUC values (proteins): COL6A3 alone: 0.74 COL6A3 + RELN: 0.83 COL6A3 + RELN + COL6A1: 0.85 | Train/test user-blinded test set | Confounding factors: body mass index (BMI), age, and neuropsychological factors | Plasma samples were available for 26 participants, including 14  symptomatic former NFL players (mean age: 56.7 years, range 46-67)  and 12 asymptomatic controls (mean age: 55.1, range 48-65) for proteomics, and 27  symptomatic former NFL players (mean age: 56.6, range 40-68)  and 25 asymptomatic controls (mean age: 57.0,  range 45-68) for ultrasensitive immunoassay for t-tau and p-tau. | Biomarker Identification | The identification of specific protein biomarkers (t-tau, p-tau181, COL6A3, RELN, COL6A1) in plasma EVs offers the possibility of developing a non-invasive blood test for CTE. This would be a major advancement, as CTE diagnosis currently relies on post-mortem brain examination. |
| 519 | Monitoring / Prognosis | Sandler *et al,* 2023 | Multiplexed Digital Characterization of Misfolded Protein Oligomers via Solid-State Nanopores | To develop a high-throughput, single-molecule method using  nanopores and DNA barcoding to accurately detect and quantify  misfolded protein oligomers, aiding in neurodegenerative disease  diagnostics and therapeutics. | Diagnostic test accuracy study | Parkinson's disease | Machine Learning | Machine learning was used in a parallel study to optimize  small-molecule inhibitors that block alpha-synuclein secondary nucleation, which in turn affects oligomer detection in nanopore assays Type of Nano: DNA nanostructure | Active learning-based docking simulations | Supervised Learning | Regression: the ML model predicted the efficacy of small  molecules in inhibiting alpha-synuclein oligomerization by modeling  their interactions with fibril surfaces | Percentage of events with an oligomer bound to the  DNA barcode: I3.08 (inhibitor): 14.4%,  Anle-138b (inhibitor): 41.8%, DMSO (control): 29.8% | Comparison with an alternative technique (micro free flow  electrophoresis, ŒºFFE) | Digital DNA barcoding was employed to label oligomer  populations for identification in multiplexed samples. | monomeric and oligomeric α-synuclein (αS) oligomers samples +  αS aggregation kinetics data | Diagnostic; Other: Therapeutic | This study offers a high-throughput, single-molecule nanopore method for detecting elusive protein oligomers, which has been demonstrated for Parkinson's drug discovery. The method surpasses existing techniques in throughput, enables in vivo oligomer quantification via click chemistry, and holds broad potential for protein misfolding and drug discovery research. |
| 578 | Monitoring / Prognosis | Tahirbegi *et al,* 2022 | Toward high-throughput oligomer detection and classification for early-stage aggregation of amyloidogenic protein | To develop a high-throughput, single-molecule photobleaching  method with machine learning to analyze the early-stage  aggregation kinetics of beta-amyloid and alpha-synuclein. | Other: Methodological Development | Alzheimer's disease; Parkinson's disease | Machine Learning | ML was used to classify fluorescence signals corresponding to different oligomeric states of proteins Type of Nano: Gold nanoparticles | Support Vector Machines (SVM) Multilayer Perceptron (MLP) Artificial Neural Networks | Supervised Learning | Classification: Classification of single-molecule fluorescence  signals to determine oligomer size. Differentiation between  monomeric and oligomeric protein states. | Multilayer Perceptron model, best model:  accuracy of 83.5% on simulated  photobleaching traces up to 19-mers | NA | Features such as initial intensity, integrated intensity,  standard deviation, kurtosis, and bleaching gradient were used. | Single-molecule photobleaching measurements  obtained through high-throughput fluorescence microscopy | Diagnostic | This study demonstrates a high-throughput single-molecule method, coupled with Machine learning, for analyzing early protein aggregation. It reveals insights into the mechanisms of Alzheimer's and Parkinson's diseases and paves the way for targeted therapies. |
| 96 | Therapy / Drug Delivery | ChandraKaushik *et al,* 2019 | Evaluation of anti-EGFR-iRGD recombinant protein with GOLD nanoparticles: synergistic effect on antitumor efficiency using optimized deep neural networks | To Investigate the frequency and prognostic significance of  EGFR alterations in glioma and develop a novel in silico deep neural network approach for screening potential EGFR inhibitors. | Other: Computational/Experimental study | Brain Cancer | Deep Learning | To analyze plasma extracellular vesicle (EV) biomarkers (tau and  β-amyloid) for predicting cognitive dysfunction in Parkinson’s disease Nanoparticle library. Type of Nano: gold nanoparticles (NPs) | Differentiable neural networks | Supervised Learning | Regression: to predict the inhibitory potential of gold nanoparticles  (AuNPs) against the EGFR receptor using an optimized  deep neural network approach. | Validation set: AUC of 0.911, accuracy of 91.3%, precision  of 90.0%, sensitivity of 100%, and specificity of 60.0% | Cross validation with previous literature | NA | The Cancer Genome Atlas (TCGA) and cBioPortal to retrieve mutational,  copy number variation, and expression data for EGFR across  different cancer types glioma and glioblastoma (GBM), combining  Low-Grade Glioma (LGG) and GBM cohorts PubChem's chemical compound library was used to  screen potential nanoparticles (NPs) | Screening; Other: EGFR can be used as an independent prognostic indicator for glioma patients | 1. In silico optimized deep neural network approach could efficiently screen a nanoparticle (NP) library for EGFR inhibition. 2. This study confirmed the potential of the combined anti-EGFR-iRGD protein and gold nanoparticles (AuNP) strategy for inhibiting tumors driven by EGFR overexpression. |
| 259 | Therapy / Drug Delivery | Kakulade *et al,* 2024 | Development, characterization and pharmacokinetic evaluation of selegiline HCl loaded cubosomal thermoreversible mucoadhesive gel for nose to brain delivery | To formulate and evaluate SGH-loaded intranasal  thermoreversible cubosomal gel to enhance its  bioavailability and ensure efficient brain targeting. | Other: Computational/Experimental study | Alzheimer's disease; Multiple sclerosis; Parkinson's disease | Machine Learning | To enhance nanoparticle formulation, drug delivery systems,  and material characterization Type of Nano: cubosomal thermoreversible mucoadhesive gel | Artificial Neural Network | Supervised Learning | Regression: to optimize the formulation of a cubosomal gel for  intranasal drug delivery using an artificial neural network to  predict particle size, entrapment efficiency, and drug release. | Best-performing formulation parameters: particle size,  entrapment efficiency, drug release, and pharmacokinetic parameters. Particle Size: 166.8 ± 3.12 nm Polydispersity Index: 0.163 ± 0.031 Zeta Potential: −20.8 ± 1.21 mV Entrapment Efficiency: 72.85 ± 1.50% In vitro Drug Release at 6 hours: 89.15 ± 1.04% Pharmacokinetic parameters in vivo: Cmax in brain: 77 ± 0.32 ng/mL area under the curve for drug concentration over time: 36.92 ± 0.41 ng.min/mL | 71.4% for training dataset 28.6% for validation dataset | They explored the use of ANN to optimize the formulation  of SGH loaded intranasal thermoreversible cubosomal gel.  However, finding a larger error than the Response Surface  Methodology model, they pursued the optimization of  the gel formulation with the latter. | one receiving intranasal administration of selegiline HCl drug  solution (1 mg/kg) and the other receiving intranasal administration of selegiline HCl-loaded cubosomal gel (1 mg/kg) Two groups: one receiving intranasal administration of selegiline  HCl drug solution (1 mg/kg) and the other receiving intranasal  administration of selegiline HCl-loaded cubosomal gel (1 mg/kg) Blood samples: via cardiac puncture at different time intervals  (30, 120, 240, and 360 minutes) + brain tissue samples 14 experimental formulations of cubosomes loaded  with Selegiline HCl | Other: Drug delivery | Although ANNs were not used to optimize gel formulation, this data-poor ML method showed competitive results to the Response Surface Methodology (RSM). The ANN model can find more complex nonlinear relationships with more data than the proposed RSM method. |
| 269 | Therapy / Drug Delivery | Karthik *et al,* 2024 | Improving brain tumor treatment with better imaging and real-time therapy using quantum dots | To introduce a comprehensive methodology merging Quantum Dots (QDs) and Real-Time Imaging-Guided Therapeutics (RIGT) to refine the precision of brain tumor radiotherapy. | Non-randomized experimental study | Brain Cancer | Deep Learning | They combine DL and nanotechnology by combining Quantum Dots (QDs) with a Real-Time Imaging-Guided Therapeutics system, integrating CNN-GAN model to improve the treatment of brain tumors. Type of Nano: near-infrared quantum dots | Hybrid Convolutional Neural Networks-Generative Adversarial Networks. CNN used: convLayer + MaxPool + convLayer + FullyConnectedLayer CNN compared: ResNet50, VGG19, AlexNet | Supervised Learning | Classification: classification for segmentation of tumor and  Regression to predict tumor size and volume (measured  in mm≥ or cm-1). | IoU (Intersection over Union): 0.89 Dice Coefficient: 0.95 F1-score: 0.94 Structural Similarity Index (SSI): 0.91 | Not used | Medical data of the patient were incorporated, to predict the evolution of the tumor and possible responses to treatment. | The distribution of the population is not precise. Still,  the following groups were used: patients who received radiotherapy, patients with brain tumors who did not receive radiotherapy,  control group without brain tumors. | Other: Treatment improvement | This study makes a breakthrough in personalized medicine by employing nanotechnology and AI methods. This is achieved with nanotechnology to be more accurate in identifying brain tumors in deep regions. Additionally, incorporating AI methods increases tumor segmentation compared to traditional CNN models. Incorporating these two methods allows real-time radiotherapy to be performed while minimizing exposure  to healthy tissue. |
| 393 | Therapy / Drug Delivery | Munteanu *et al,* 2021 | Prediction of Anti-Glioblastoma Drug-Decorated Nanoparticle Delivery Systems Using Molecular Descriptors and Machine Learning | To develop a computational model for predicting the formation  of drug-decorated nanoparticle delivery systems with  anti-glioblastoma activity using molecular descriptors and  machine learning techniques | Other: Computational/Experimental study | Brain Cancer | Machine Learning | The ML were trained to predict the likelihood of nanoparticle-drug complexes forming with anti-glioblastoma properties Type of Nano: Drug-decorated nanoparticles (DDNPs) | Bagging Classifier Decision Tree Classifier Random Forest Classifier XGBoost Classifier Gradient Boosting Classifier K-Nearest Neighbors Gaussian Naive Bayes Logistic Regression Linear Discriminant Analysis AdaBoost Classifier | Supervised Learning | Classification: predicting whether a drug-nanoparticle complex  has anti-glioblastoma properties. Identifying key molecular  descriptors affecting prediction performance | For the best model - Bagging Classifier: F1-Score: 0.87 AUROC: 0.96 | 75% training, 25% test | Feature selection reduced 104 molecular descriptors to  41 important features Molecular descriptors were transformed based on  perturbation theory to improve predictive power | 855,129 drug-nanoparticle complexes (combination of  nanoparticle experimental data with drug assay data  from the ChEMBL database and literature sources) molecular descriptors of both drugs and nanoparticles their perturbations under various experimental conditions For Drug, Polar Surface Area (PSA) and logP For Nanoparticle, surface area,  Van der Waals volume, and coating properties | Other: Therapeutics | The PTML model provides a powerful tool for the virtual screening of drug-nanoparticle combinations, significantly speeding up the identification of promising candidates for glioblastoma treatment. |
| 570 | Therapy / Drug Delivery | Sun *et al,* 2021 | Bionanoscale Recognition Underlies Cell Fate and Therapy | Quantify and compare the bionanoscale recognition of 1T-MoS2  (octahedral coordination) and 2H-MoS2 (triangular prism coordination)  with fibronectin and liposomes | Other: Computational/Experimental study | Parkinson's disease | Machine Learning | Machine learning models, particularly Random Forest (RF) and  Structural Equation Models (SEMs), are used to analyze the  influence of nanostructured architectures on cell viability,  adhesion, and differentiation. These models help identify key morphological features affecting cell health Type of Nano: 1T-MoS2 (octahedral coordination) and 2H-MoS2  (triangular prism coordination) nanostructures | Random Forest Model Structural Equation Model | Supervised Learning | Classification: RF Model: Classifies cell health based on  morphological features like neurite length and cell area. Regression: SEM Model: Quantifies how specific  nanostructured architectures enhance cell viability. | Accuracy of RF Model: 89.2% AUC of RF Model: 0.893 | NA | Feature extraction was performed on seven morphological metrics related to neurite  outgrowth and cell health. | in vitro experiments using PC12 cells, a neuronal cell line Parkinson’s disease (PD) model mouse | Other: Cell-based Therapeutics | This study demonstrates that octahedral coordination in nanomaterials, specifically 1T-MoS2, significantly enhances bio-nanoscale recognition, improving cell fate control and therapeutic potential. This discovery enables the rational design of biomaterials for targeted drug delivery, cell therapy, and neurodegenerative disease treatment, particularly Parkinson's disease, by modulating interactions with key biomolecules like fibronectin, liposomes, and alpha-synuclein. |
